# Stabilizing Atomically Dispersed Au With Adjacent Pt for Spatially Precise Molecule Recognition

**DOI:** 10.1002/advs.74720

**Published:** 2026-03-09

**Authors:** Rui Tang, Xiangyu Xiao, Jingyi Yao, Qing Wang, Qijing Gao, Nan Zhou, Fanyi Kong, Dingguan Liang, Kangning Dong, Liang Xiong, Yuwei Cao, Zhendong Lei, Liang Tang

**Affiliations:** ^1^ Key Laboratory of Organic Compound Pollution Control Engineering (MOE) School of Environmental and Chemical Engineering Shanghai University Shanghai P. R. China; ^2^ College of Environmental Science and Engineering Tongji University Shanghai P. R. China; ^3^ State Key Laboratory of Tribology Department of Mechanical Engineering Tsinghua University Beijing P. R. China

**Keywords:** atomically dispersed Au catalysts, multi‐centered active sites, platinum group metals, Pt‐induced stabilization, spatially precise recognition

## Abstract

Atomically dispersed Au catalysts provided well‐defined active sites but are constrained by poor structural stability and insufficient spatially resolved interactions with structurally complex multi‐functionalized molecules (SCMMs), limiting their applicability in selective catalysis. Herein, a generalizable adjacency‐assisted strategy to stabilize Au sites and enable multi‐site recognition toward SCMMs by introducing Pt atoms into defect‐rich CeO_2_ supported Au single atoms (Au_1_Pt_1_‐CeO_2_) is proposed. Experimental and computational analyses demonstrate that Pt incorporation suppresses metallic Au° formation by strengthening Au‐O orbital coupling and inducing electronic redistribution across Au‐O‐Ce bridge, thereby preventing Au atom aggregation and facilitating interfacial charge transfer. The multi‐centered configuration creates cooperative interaction sites in which Ce, Au, and Pt preferentially coordinate with the F, ‐COOH, and ‐C = O moieties of norfloxacin (NOR), respectively, establishing a well‐defined adsorption geometry that enhances electrochemical catalytic performance and enables spatially precise recognition toward SCMMs. Extending this strategy to other platinum group metals (PGMs = Ir, Pd) confirms a potentially generalizable stabilization effect driven by suitable *d*‐orbital electronic modulation, whereas other transition metal atoms (Fe, Ag) fail to induce similar behavior. This work provides a general interfacial modulation perspective that may inspire the rational development of robust and recognition‐oriented catalysts for SCMMs.

## Graphical abstract

A strategy to stabilize atomically dispersed Au catalysts by introducing Pt atoms into CeO_2_ supports is presented. Pt incorporation prevents Au aggregation, enhances Au‐O orbital coupling, and enables precise recognition of multi‐functionalized molecules, improving catalytic performance and the structural stability of Au‐based catalysts in electrochemical reactions.

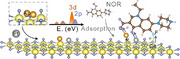

## 1 Introduction

Atomically dispersed Au catalysts have attracted extensive attention due to their high atomic utilization efficiency and well‐defined coordination environments [1, 2, 3, 4, 5]. In particular, Au‐oxide single‐atom catalysts (SACs) benefit from the distinctive 5d orbital configuration of Au, which facilitates effective electronic interactions and interfacial confinement with oxide supports, synergistically enhancing catalytic activity and selectivity [6, 7, 8, 9, 10]. However, the high surface energy of isolated Au atoms renders them thermodynamically unstable, often resulting in migration and aggregation into nanoclusters or nanoparticles, accompanied by a loss of catalytic efficiency and site specificity [11, 12, 13]. Moreover, their intrinsically simple electronic structures of single Au sites generally lead to monofunctional adsorption behavior, restricting their ability to interact with structurally complex multi‐functionalized molecules (S+CMMs), and confining them mainly to relatively simple small‐molecule catalytic processes, such as CO_2_ reduction [14], CO oxidation [15], and the oxygen reduction reaction (ORR) [16].

For SCMMs, such as complex organic compounds containing aromatic or heterocyclic structures, achieving efficient catalytic performance remains challenging due to their large molecular size [17], multiple functional groups [18], and significant steric hindrance [19], which collectively lead to weak adsorption [20], low selectivity [21, 22], and inefficient charge transfer [23]. In addition, the rigid aromatic rings present in many SCMMs restrict conformational flexibility and hinder favorable spatial alignment with mononuclear active sites. The coexistence of diverse functional groups further promotes nonspecific interactions with the catalyst surface, resulting in signal interference and reduced selectivity and accuracy [24, 25]. Therefore, it is essential to develop advanced atomically dispersed Au catalysts that can overcome these structural and electronic constraints to achieve stable, efficient, and selective catalysis of SCMMs.

For SACs with high migration tendency and simple electronic structures, doping heteroatoms or constructing dual‐metal sites is considered a viable approach to enhance stability and catalytic efficiency effectively [26, 27, 28]. For instance, Zhang et al. adopted a complex‐assisted pyrolysis strategy to introduce Cu and construct a Co/Cu‐N‐C bimetallic catalyst, which not only boosted ORR activity but also significantly improved stability due to the constraining effect of the Cu‐N supported on the Co sites [29]. Xiao et al. developed Fe‐N‐C‐supported Pt nanoparticle electrocatalysts and found that Fe and N doping suppressed Pt surface oxidation and dissolution by negatively shifting the Pt *d*‐band center, thereby reducing the tendency of Pt particle agglomeration [30]. In addition, incorporating Pt can further enhance catalytic performance owing to its unique electronic structure and superior intrinsic activity. Yin et al. demonstrated that PtAu nanoparticles, formed by alloying Pt with the more electronegative Au, exhibited efficient synergistic catalysis in ethanol oxidation and hydrogen evolution. The alloy modulates the Pt *d*‐band center, optimizing intermediate adsorption and thus improving catalytic activity and selectivity [31]. Besides, the interfacial synergy between oxide supports and metal atoms can impart adaptive stability. Defect‐rich CeO_2_ (V‐CeO_2_) provides a tunable platform, as the loaded metal‐induced modulation of Ce 4f electrons promotes Ce^3+^ and oxygen vacancy (O_V_) formation [32], generating multi‐center active sites with enhanced surface reactivity and adsorption capacity.

However, existing approaches to enhance primarily focus on structural durability or catalytic performance, but rarely achieve simultaneous stability improvement and spatially defined multi‐site recognition toward SCMMs, leaving a critical gap in the rational design of atomically dispersed catalysts that couple stabilization with site‐specific interation. To bridge this gap, we propose a generalizable adjacency‐assisted strategy designed to couple atomic stability with spatially selective multi‐site recognition. Positioning atomically dispersed Pt atoms adjacent to isolated Au atoms on V‐CeO_2_ (Au_1_Pt_1_‐CeO_2_) generates multi‐centered sites that simultaneously strengthen Au‐O interfacial orbital coupling to suppress aggregation and create cooperative multi‐center interaction sites capable of engaging distinct functional groups within SCMMs. This catalyst exhibits remarkable catalytic performance toward norfloxacin (NOR), a representative SCMM characterized by a rigid polycyclic framework and multifunctional groups, including a chelating N‐methylpiperazine moiety and fluorine atoms. Such structural complexity imposes significant steric hindrance and adsorption challenges. Pt‐induced stabilization in Au_1_Pt_1_‐CeO_2_ promotes cooperative electronic rearrangement, enabling synergistic interactions among Au, Pt, and Ce at spatially distributed active centers with key molecular moieties in NOR, facilitating site‐specific molecular recognition and driving efficient electrochemical catalytic reduction of NOR [33, 34, 35, 36, 37] Furthermore, we extend this strategy to other platinum‐group metals (PGM = Ir, Pd) and non‐PGM elements (Ag, Fe), confirming the generalizability of the stabilization effect driven by suitable *d*‐orbital electronic modulation. This work offers a mechanistic perspective on enhancing stability and achieving precise functional‐group recognition in SCMMs.

## 2 Results and Discussion

### 2.1 Material Morphology Characterization and Structural Analysis

**FIGURE 1:**
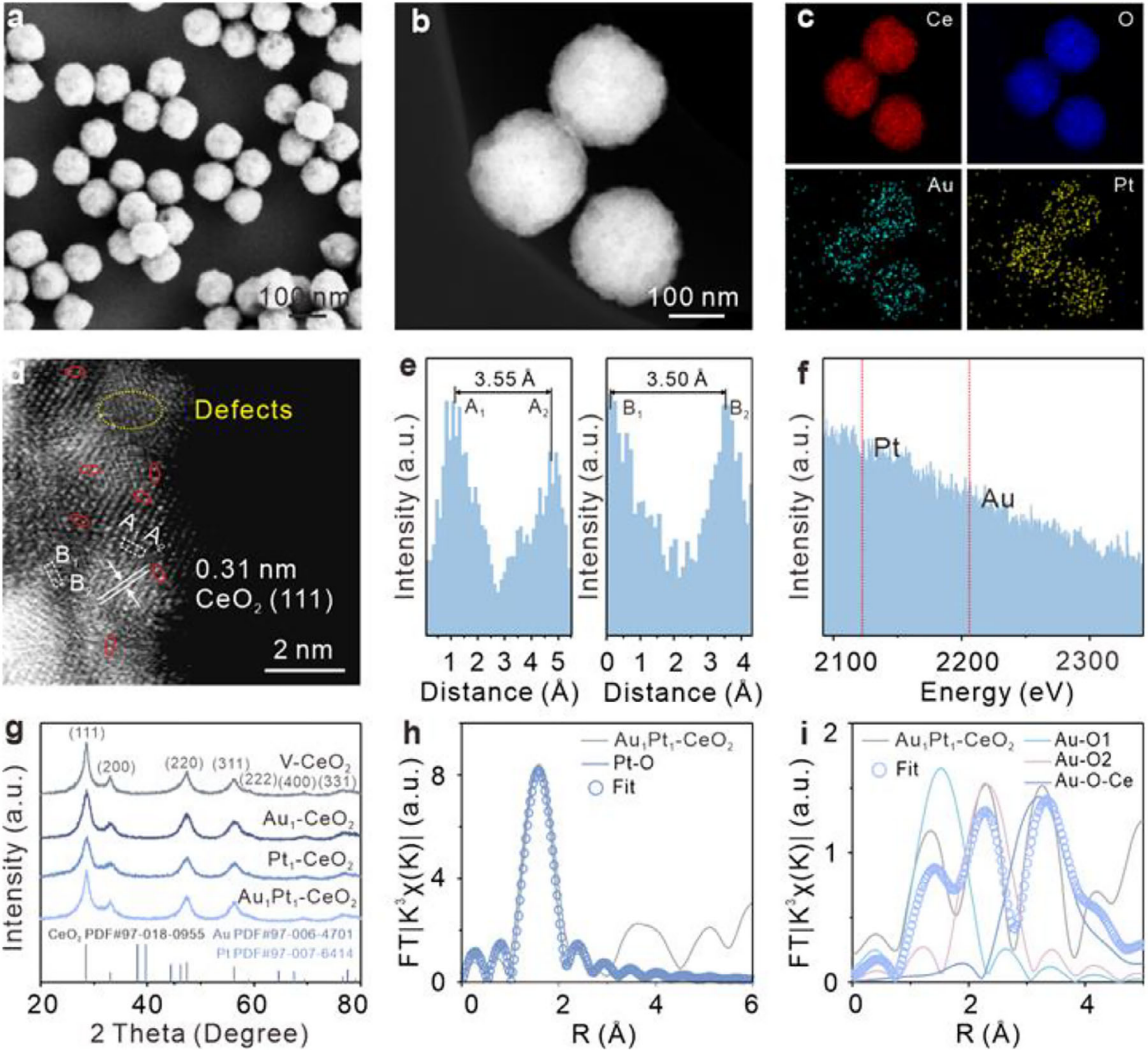
Morphology and structure characterizations of Au_1_Pt_1_‐CeO_2_. (a) SEM image of Au_1_Pt_1_‐CeO_2_. (b) HAADF‐STEM image of Au_1_Pt_1_‐CeO_2_. (c) Element mapping of Ce (red), O (blue), Au (cyan), and Pt (yellow) shows a uniform distribution, corresponding to Figure 1b. (d) High magnification of HAADF‐STEM reveals that Au and Pt are atomically dispersed on the V‐CeO_2_ surface as isolated single atoms without aggregation. Pairs of atoms with extremely low loading close to 1:1 are circled in red; the defects are circled by the dotted yellow line. (e) The corresponding intensity profiles for zones A and B in Figure 1d. (f) Point scan EELS spectra of Au_1_Pt_1_‐CeO_2_. (g) XRD spectra of V‐CeO_2_, Au_1_‐CeO_2_, Pt_1_‐CeO_2_, and Au_1_Pt_1_‐CeO_2_, confirming the absence of metal peaks. (h) Pt L_3_‐edge EXAFS spectra (k^3^‐weighted) of Au_1_Pt_1_‐CeO_2_ confirm that Pt is O‐bridged on the V‐CeO_2_ surface without the presence of metallic Pt. (i) EXAFS spectra (k^3^‐weighted) of Au_1_Pt_1_‐CeO_2_ in R‐space at the Au L_3_‐edge show that Au is loaded on the V‐CeO_2_ surface by O‐bridging.

The morphological and structural properties of the synthesized V‐CeO_2_, Pt_1_‐CeO_2_, Au_1_‐CeO_2_, and Au_1_Pt_1_‐CeO_2_ were systematically characterized, as shown in Figure 1 and Figures S1–S4. Scanning electron microscopy (SEM) images (Figure 1a) and the high‐angle annular dark‐field scanning transmission electron microscopy (HAADF‐STEM) images indicate that the V‐CeO_2_ support maintained its nanosphere morphology with an average diameter of approximately 150 nm and rough surfaces, following the loading of Au and Pt single atoms (Figure 1b). Elemental mapping of Au_1_Pt_1_‐CeO_2_ (Figure 1c), corroborated by energy dispersive spectroscopy (EDS) analysis (Figure S4c), demonstrates a homogeneous distribution of Ce, O, Au, and Pt, with no detectable impurities. HAADF‐STEM image displays bright atomic‐scale spots on the V‐CeO_2_ surface (Figure 1d), corresponding to isolated Au and Pt atoms, confirming the successful synthesis of the dual‐atom catalyst (DAC) [38, 39]. Oxygen defects in V‐CeO_2_ are clearly visible and highlighted by yellow dashed circles. No morphological differences are observed for Au_1_‐CeO_2_ and Pt_1_‐CeO_2_, suggesting the structural robustness of the V‐CeO_2_ support. EDS spectra confirm that both Au_1_‐CeO_2_ and Pt_1_‐CeO_2_ display clear signals for their respective elemental components. Elemental distribution mapping further verifies the uniform dispersion. High‐magnification HAADF‐STEM images directly visualize the atomic dispersion of Au and Pt without nanoparticle aggregation.

Figure 1e presents the elemental intensity profiles for regions A and B in Figure 1d, indicating an interatomic distance of about 3.50 Å between adjacent metal atoms, consistent with this, statical analysis of multiple diatomic sites shows that most distance fall in the range of 3.50–3.70 Å (Figure S5). Electron energy loss spectroscopy (EELS) on the diatomic sites further confirms the atomic‐scale proximity of Au and Pt atoms (Figure 1f), confirming the presence of Au and Pt diatomic sites. X‐ray diffraction (XRD) patterns for Au_1_‐CeO_2_, Pt_1_‐CeO_2_, and Au_1_Pt_1_‐CeO_2_ display no detectable metallic diffraction peaks (Figure 1g), excluding the formation of large nanoparticles. Inductively coupled plasma (ICP) analysis quantifies the Au and Pt contents (Table S1). Au_1_Pt_1_‐CeO_2_ contains 0.79 wt.% Au and 0.76 wt.% Pt, respectively, nearly a 1:1 ratio, closely matching the loading of Au (0.69 wt.%) and Pt (0.76 wt.%) loadings in single atom (SA) samples.

To further clarify the localized spatial structure of the dual‐site structure, extended X‐ray absorption fine structure (EXAFS) spectroscopy is performed at the Pt L_3_‐edge and Au L_3_‐edge for the Au_1_Pt_1_‐CeO_2_ (Figure 1h,i, Tables S2 and S3), revealing a prominent Pt‐O scattering path at approximately 1.60 Å with a coordination number 8.47, while no Pt‐Pt scattering path is observed. It suggests that Pt species are stabilized as single atoms anchored on V‐CeO_2_ via Pt‐O bonds, confirming a highly dispersed state. The EXAFS spectrum at the Au L_3_‐edge of Au_1_Pt_1_‐CeO_2_ (Figure 1i), displays pronounced peaks near 1.70 Å and 2.10 Å, corresponding to Au‐O scattering paths, indicative of two coordination environments, and the non‐agglomeration of Au on V‐CeO_2_ surface [40]. Further fitting results show that Au is coordinated with about four O atoms, with average bond lengths of 1.95 and 2.77 Å. The fitting curves in Figure S6 are well fitted to the original data, supporting the accuracy and reliability of the above analyses.

### 2.2 Electronic Structure and Local Coordination Analysis

**FIGURE 2:**
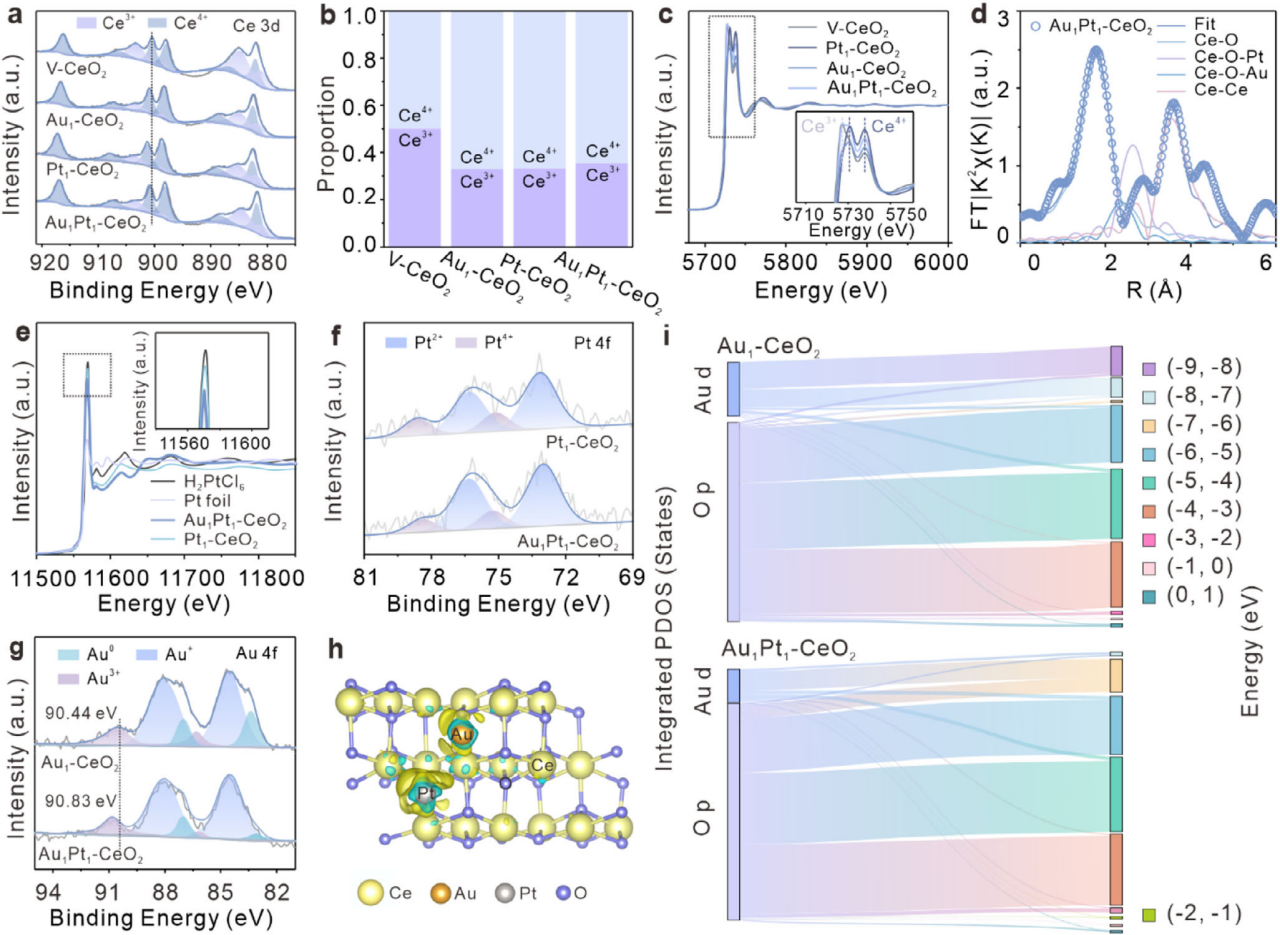
Coordination environment characterizations. (a) XPS of Ce 3d in V‐CeO_2_, Au_1_‐CeO_2_, Pt_1_‐CeO_2_, and Au_1_Pt_1_‐CeO_2_, and (b) their corresponding Ce^3+^ to Ce^4+^ ratio histogram. (c) Normalized Ce L_3_‐edge XANES spectra and magnified image (inset) of V‐CeO_2_, Pt_1_‐CeO_2_, Au_1_‐CeO_2_, and Au_1_Pt_1_‐CeO_2_. (d) Ce L_3_‐edge EXAFS spectra in R space (k^2^‐weighted) and the fitting curves of Au_1_Pt_1_‐CeO_2_. (e) Normalized Pt L_3_‐edge XANES spectra and magnified image (inset) of H_2_PtCl_6_, Pt foil, Au_1_Pt_1_‐CeO_2_, and Pt_1_‐CeO_2_, where the Pt valence state changes indicate an interaction between Au and Pt. (f) XPS of Pt 4f in Pt_1_‐CeO_2_ and Au_1_Pt_1_‐CeO_2_, and (g) XPS of Au 4f in Au_1_‐CeO_2_ and Au_1_Pt_1_‐CeO_2_, verify the atomic dispersion of Au/Pt and their electron interaction, synergistically enhancing redox properties. (h) The differential charge density image of Au_1_Pt_1_‐CeO_2_, showcasing the electron transfer behavior among the elements in Au_1_Pt_1_‐CeO_2_, is consistent with the XPS analysis results. (i) Sankey diagram illustrating the allocation of electronic states from Au d and O p orbitals to different energy intervals in Au_1_‐CeO_2_ and Au_1_Pt_1_‐CeO_2_. The width of each flow is proportional to the integrated PDOS value (in states).

The X‐ray photoelectron spectroscopy (XPS) was employed to investigate the surface chemical states and electronic interactions of the catalysts. XPS of the Ce 3d spectra identified distinct oxidation states resulting from oxygen defects: peaks at 881.15, 884.76, 898.50, and 903.20 eV are assigned to Ce^3+^ species, while those at 882.06, 886.40, 897.95, 900.40, 906.20, and 916.15 eV correspond to Ce^4+^ (Figure 2a) [41]. Notably, the total Ce 3d profiles of Au_1_‐CeO_2_, Pt_1_‐CeO_2_, and Au_1_Pt_1_‐CeO_2_ exhibit positive binding energy shifts of 0.50, 0.55, and 0.40 eV, respectively, compared to the V‐CeO_2_ support, indicating electron transfer from Ce upon Au and Pt incorporation. The larger shift in Pt_1_‐CeO_2_ may indicate more pronounced Pt‐O interaction than Au‐O interaction in Au_1_‐CeO_2_, which will be discussed in subsequent sections. The reduced shift in Au_1_Pt_1_‐CeO_2_ reflects a coupled Au/Pt‐O‐Ce interface where Pt‐induced Au‐support coupling leads to interfacial charge redistribution and partial compensation of Ce electron depletion [42, 43]. Quantitative analysis of the Ce^3+^ concentration, calculated as [Ce^3^^+^] = S(Ce^3^^+^)/[S(Ce^3^^+^) + S(Ce^4^^+^)] (where S represents integrated XPS peak areas), reveals a marked decrease from 50.10% in V‐CeO_2_ to 33.20% in Au_1_‐CeO_2_, 33.30% in Pt_1_‐CeO_2_, and 35.50% in Au_1_Pt_1_‐CeO_2_ (Figure 2b). This reduction correlates with the diminished O_v_ concentration observed in O 1s XPS and EPR signal spectra, probably attributed to the occupation of O_v_ sites by anchored Au and Pt (Figures S7–S9) [44, 45, 46].

Figure 2c compares the normalized Ce L_3_‐edge XANES spectra of catalysts. Relative to V‐CeO_2_, the metal‐loaded catalysts exhibit an enhancement in the white line peaks, indicating an increase in Ce^4+^ content [47], which aligns with the Ce 3d XPS results. The distinct Ce^3+^ peaks at low energies, attributed to the dipole‐allowed hopping from Ce 2p to Ce 5d, are more pronounced in Au_1_Pt_1_‐CeO_2_ than in the SA counterparts [48, 49]. This electronic excitation of the Ce 5d orbital can enhance the electron transfer ability of Au_1_Pt_1_‐CeO_2_ samples. The Ce L_3_‐edge (k^3^‐weighted) R‐space EXAFS analysis provides insight into the local coordination. For V‐CeO_2_, two Ce‐O scattering paths are identified: Ce‐O1 at ∼1.80 Å, unaffected by O_V_, and Ce‐O2 at 3.15 Å with a reduced coordination number (CN = 1.66), possibly due to localized lattice expansion induced by O_V_ (Table S4) [50]. After the incorporation of Au/Pt, newly emerging Ce‐O‐M (M = Au, Pt) scattering paths are observed at 3.17 Å (CN = 1.80) and 3.19 Å (CN = 1.66), respectively. These metal‐specific bond lengths significantly exceed the intrinsic Ce‐O distance in V‐CeO_2_, indicating that the isolated Au/Pt atoms are anchored on the V‐CeO_2_ surface via oxygen bridges, resulting in bond length elongation.

According to the Ce L_3_‐edge EXAFS spectra of Au_1_Pt_1_‐CeO_2_ in Figure 2d, two coexisting Ce‐O‐M (M = Au, Pt) scattering paths are observed. The scattering peak of Ce‐O‐Pt exhibits higher intensity than Ce‐O‐Au, likely caused by a stronger degree of hybridization between Pt *d*‐electrons and O 2p orbitals. The higher coordination number of Ce‐O‐M in Au_1_Pt_1_‐CeO_2_ (CN = 2.10 for Ce‐O‐Au, CN = 2.30 for Ce‐O‐Pt) compared to the SA samples (CN = 1.80 for Au_1_‐CeO_2_, CN = 1.66 for Pt_1_‐CeO_2_) suggests a synergistic dispersion effect between Au and Pt that jointly inhibits their aggregation [51]. The PDF refinement results show increased peak areas of Au‐O, Pt‐O, and Ce‐O at 2.30 Å in Au_1_Pt_1_‐CeO_2_ (Figure S10), further confirming the strengthened M‐O interactions. Meanwhile, the 0.02 Å contraction of the Ce‐O length in Au_1_Pt_1_‐CeO_2_ corroborates the regulatory effect of dual atomic loading on lattice stress (Table S4), which is conducive to improving the structural stability of Au_1_Pt_1_‐CeO_2_. Additional details are provided in Figures S11 and S12.

The normalized Pt L_3_‐edge XANES spectra show that the valence states of Pt in Pt_1_‐CeO_2_ and Au_1_Pt_1_‐CeO_2_ are between 0 and +4. The XPS Pt 4f spectra presented in Figure 2f further confirm a decrease in the relative content of Pt^4+^ in Au_1_Pt_1_‐CeO_2_ [52], which suggests that the interaction between Au and Pt facilitates greater electron acceptance by Pt single atoms, consistent with the EXAFS results. Figure 2g demonstrates a marked reduction in the content of Au^0^ species in Au_1_Pt_1_‐CeO_2_, while Au^3+^ exhibits a positive binding energy shift relative to Au_1_‐CeO_2_. It indicates that Pt modulates the electron cloud density distribution of Au. Simultaneously, complementary Pt charge modulation collectively enhances the overall redox capacity of Au_1_Pt_1_‐CeO_2_. In conjunction with the structural characterization results, we conduct DFT calculations to determine optimized atomic configurations and calculate the formation energy of these configurations (Figure S13). The result shows that the Au_1_Pt_1_‐CeO_2_ dual‐atom configuration, in which Au and Pt occupy O‐sites in a relatively tilted position closer to O atoms, exhibits the lowest formation energy (E_f_ = ‐5.40 eV), whereas both Au‐CeO_2_ and Pt‐CeO_2_ exhibit higher E_f_ (Figure S13), indicating reduced stability. Differential charge density analysis reveals that Au can modify the electron cloud distribution around Pt compared to Pt‐CeO_2_ (Figure 2h; Figure S14) and mitigate electron loss, consistent with the XPS results. The electron enrichment at Pt sites enhances catalytic reduction activity.

To assess the impact of defect states, the band structure diagram illustrates that O_v_ in V‐CeO_2_ introduces localized states near the Fermi level (E_fer_), mainly originating from Ce 4f orbitals (Figure S15). The increased electron density around the E_fer_ improves the conductivity of V‐CeO_2_ compared to pristine CeO_2_ [53]. The projected density of states (PDOS) diagram confirms that the incorporation of Au and Pt atoms increases the Ce 3d electron density near the E_fer_ (Figure S16), thereby improving the electronic conductivity. It is noted that Pt d hybridization with O p in Pt_1_‐CeO_2_ is more pronounced than Au‐O interactions in Au_1_‐CeO_2_, supporting that Pt‐O interactions may stronger than Au‐O. Furthermore, compared to Au/Pt SACs, both the Au and Pt *d*‐band centers in Au_1_Pt_1_‐CeO_2_ exhibit positive shifts, suggesting superior catalytic activity [54]. Moreover, Pt incorporation strengthens Au‐O interactions, as evidenced by the emergence of new electronic states within −2.00 to −1.00 eV energy range (Figure 2i). This reinforced orbital coupling is identified as the electronic‐level origin of the Pt‐induced stabilization, effectively pinning the Au atom to the oxide support and suppressing its immigration.

### 2.3 Electrochemical Catalytic Performance Investigation

**FIGURE 3:**
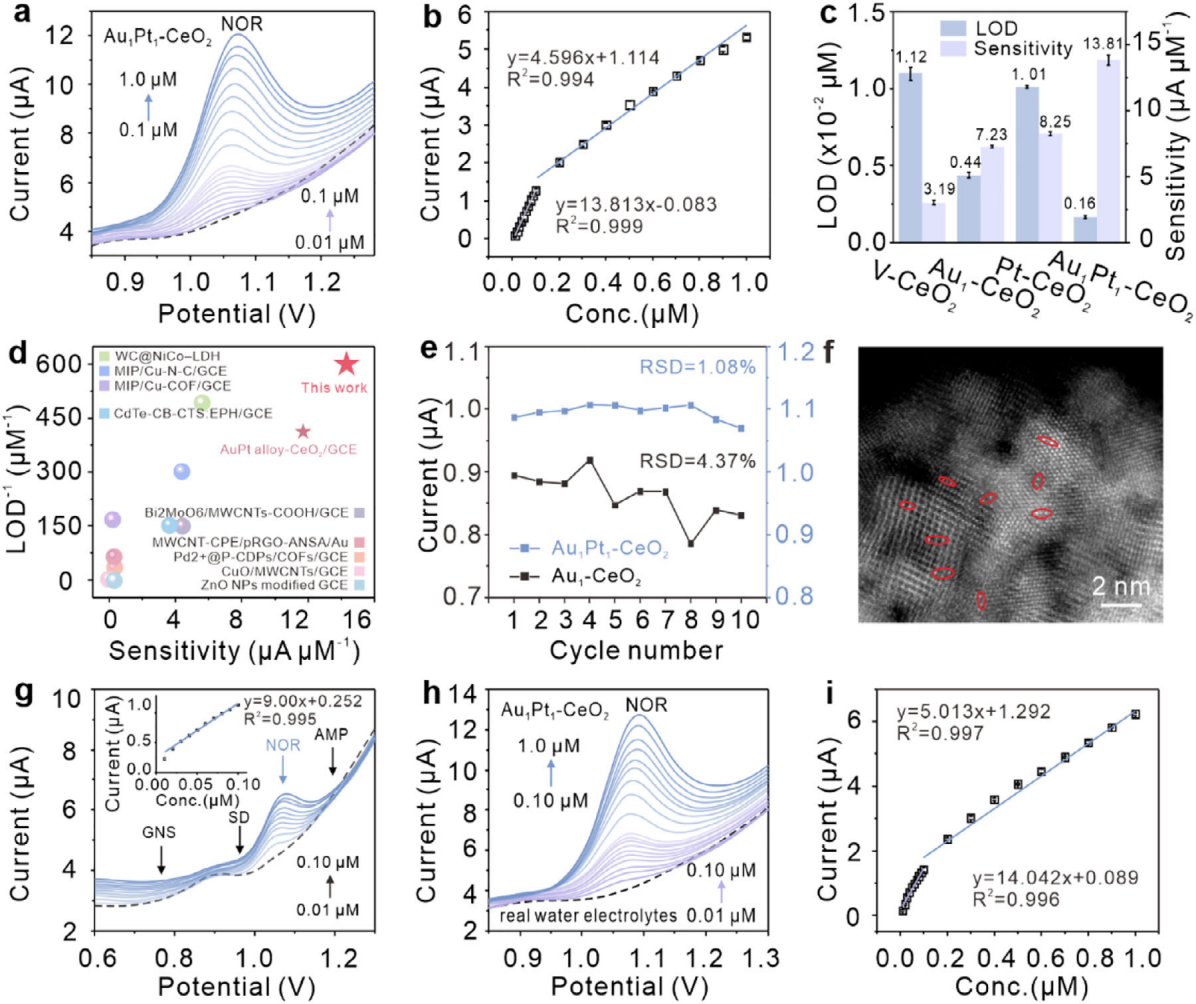
The electrochemical catalytic performance of Au_1_Pt_1_‐CeO_2_ toward NOR. (a) SWV response signals of Au_1_Pt_1_‐CeO_2_ toward NOR at extremely low concentration (0.01–0.10 µM, 10 steps with 0.01 µM intervals) and low concentration (0.10–1.0 µM, 10 steps with 0.10 µM intervals). (b) Excellent linear correlations between peak currents and NOR concentrations of Au_1_Pt_1_‐CeO_2_ for NOR detection in the range of 0.01–0.10 µM (R^2^ = 0.999) and 0.10–1.00 µM (R^2^ = 0.994). Repeated 3 times. (c) Comparisons of sensitivities and LODs of V‐CeO_2_, Au_1_‐CeO_2_, Pt_1_‐CeO_2_, and Au_1_Pt_1_‐CeO_2_ modified electrodes for NOR detection. (d) The sensitivity and LOD of Au_1_Pt_1_‐CeO_2_ and AuPt alloy‐CeO_2_ toward NOR outperform other metal‐based catalysts or metal‐oxide catalysts previously reported in literature. (e) 10 continuous electrochemical experiments at 0.05 µM NOR show that Au_1_Pt_1_‐CeO_2_ (RSD = 1.08% < 5%) exhibits excellent stability with little current fluctuation, superior to Au_1_‐CeO_2_ (RSD = 4.37%). (f) The HAADF‐STEM image indicates that AuPt dual atoms demonstrate no aggregation after the experiments of Figure S23. Pairs of atoms were circled in red. (g) Anti‐interference experiments of 0.01‐0.10 µM NOR detected by Au_1_Pt_1_‐CeO_2_ in the coexistence of 1.0 µM AMP, SD, and GNS, which belong to distinct pharmacological classes of antibiotics. The illustration shows the corresponding well linear correlations between peak currents and NOR concentrations. (h) SWV response of Au_1_Pt_1_‐CeO_2_ to NOR in real water electrolyte performs an extremely low (0.01–0.10 µM, R^2^ = 0.996) and low (0.10–1.0 µM, R^2^ = 0.997) concentration ranges, each with 10 data points measured in triplicate at uniform intervals. The corresponding linear equations are shown in (i).

The homogeneous aqueous solution of the as‐synthesized catalysts was drop‐coated onto the glassy carbon electrode (GCE) surface for preliminary electrochemical evaluation. Cyclic voltammetry (CV) and electrochemical impedance spectroscopy (EIS) results (Figure S17) indicate that the Au_1_Pt_1_‐CeO_2_/GCE exhibits the best electrochemical conductivity compared with the others, as evidenced by its lowest charge transfer resistance (R_et_ = 42.00 Ω) and highest apparent electron transfer rate. Under the optimal experimental conditions with a three‐electrode system (GCE, Pt wire counter electrode, Ag/AgCl reference electrode) in 0.10 M HAc‐NaAc buffer (Figure S18), square wave voltammetry (SWV) was employed to investigate the electrochemical catalytic performance of NOR within a concentration range of 0.01–1.00 µM. As shown in Figure 3a, distinct oxidation peaks appear at about +1.08 V, with current intensities that correlate positively with NOR concentrations. The linear calibration plot is obtained (R^2^ = 0.999, Figure 3b), from which the sensitivity and limit of detection are derived (LOD = 0.0016 µM, calculated by 3σ/S method). The sensitivity reaches 13.81 µA µM^−1^ in the low concentration range of 0.01–0.10 µM, and 4.60 µA µM^−1^ in the concentration range of 0.10–1.00 µM. The higher sensitivity at low concentrations (0.01–0.10 µM) is attributed to unsaturated active sites, whereas at higher concentrations, partial site saturation limits catalytic accessibility, attenuating the slope of the linear response. Here, σ represents the standard deviation of blank measurements while S denotes the linear regression slope.

Comparative analysis with V‐CeO_2_ (3.19 µA µM^−1^), Au_1_‐CeO_2_ (7.23 µA µM^−1^), Pt_1_‐CeO_2_ (8.25 µA µM^−1^), a physical mixture of Au_1_‐CeO_2_ and Pt_1_‐CeO_2_ (denoted as Au_1_Pt_1_‐CeO_2_‐pm, 7.01 µA µM^−1^), and AuPt alloy‐CeO_2_ (11.86 µA µM^−1^, Figures S19–S22) modified electrodes reveals that the Au_1_Pt_1_‐CeO_2_/GCE delivers the lowest LOD and highest sensitivity, especially in the low concentration range (Figure 3c). Compared to V‐CeO_2_ and single metal‐loaded materials, V‐CeO_2_ itself exhibits relatively limited catalytic activity and has not effectively enhanced the electrocatalytic performance toward NOR. Only with the introduction of Au and Pt was a significant increase in activity achieved, particularly in terms of enhanced sensitivity at low concentrations, further demonstrating the substantial promotional effect of metal loading on the catalytic performance of V‐CeO_2_. Furthermore, compared with previously reported metal‐ or metal oxide‐based electrochemical sensors [55, 56, 57, 58, 59, 60, 61, 62, 63] Au_1_Pt_1_‐CeO_2_/GCE presents exceptional NOR catalytic performance (Figure 3d), which can be ascribed to the synergistic electronic effects between AuPt dual‐atom sites and the V‐CeO_2_ support. It is worth noting that the AuPt‐alloy catalysts may also have synergistic effects, but their random atomic arrangements and lack of well‐defined, spatially separated active sites result in heterogeneous adsorption configurations and imprecise targeting toward NOR, leading to inferior electrochemical performance. To evaluate its electrochemical stability, the Au_1_Pt_1_‐CeO_2_/GCE and Au_1_‐CeO_2_/GCE are subjected to 10 consecutive SWV cycles in a HAc‐NaAc buffer solution containing 0.05 µM NOR (Figure 3e). The relative standard deviation (RSD) of 1.08% (<5%) for Au_1_Pt_1_‐CeO_2_ is markedly lower than that of Au_1_‐CeO_2_ (RSD = 4.37%), which demonstrates that the presence of Pt SAs can enhance the electrochemical stability significantly. Extending the number of cycles to 30 under 0.5 µM NOR revealed that Au_1_Pt_1_‐CeO_2_/GCE still exhibited satisfactory stability (RSD = 4.35%). Further details are provided in Figures S23 and S24.

HAADF‐STEM characterization (Figure 3f) of the post‐tested Au_1_Pt_1_‐CeO_2_ catalyst confirms that the Au‐Pt dual atoms (red circle) remain atomically dispersed on V‐CeO_2_ without aggregation during the redox reaction. The reproducibility and selectivity of Au_1_Pt_1_‐CeO_2_ toward NOR are validated in Figures S25 and S26. Testing against other SCMMs, including three high‐concentration antibiotics from different classes, ampicillin (AMP), sulfadiazine (SD), and gentamicin sulphate (GNS), which respectively contain a β‐lactam ring and an amino group, a sulfonamide group attached to a benzene ring, and an aminocyclitol core linked to amino sugar moieties, revealed no response signals. Additional comparative selectivity tests using Au_1_‐CeO_2_, Pt_1_‐CeO_2_ and AuPt alloy‐CeO_2_ were further performed (Figures S27–S29). These comparisons provide further support that the selectivity is closely associated with the local Au‐Pt adjacency in Au_1_Pt_1_‐CeO_2_, in line with the intended diatomic configuration. When these antibiotics were used as coexisting interferents (Figure 3g), there are no obvious response signals of Au_1_Pt_1_‐CeO_2_/GCE at the peak positions of about +1.00 V, +0.70 V, and +1.25 V for SD, GNS, and AMP, indicating that Au_1_Pt_1_‐CeO_2_ exhibits exceptionally high selectivity for NOR. The oxidation peak current slightly decreases under the coexistence of interfering antibiotics at a concentration of 100 times the NOR concentration, which may be due to the interfering antibiotics occupying a small number of active sites on the surface of Au_1_Pt_1_‐CeO_2_. Still, it maintains a robust overall response, indicating excellent anti‐interference capability, which is clearly superior to that of Au_1_‐CeO_2_, Pt_1_‐CeO_2_, and AuPt alloy‐CeO_2_ (Figure S30).

For practical validation, a water sample and sediment from the Suzhou Creek in Shanghai were collected for electrochemical catalysis (Figure 3h,i; Figure S31). A 0.22 µm membrane filter was utilized to filtrate river water samples to remove particulate matter. To enhance the conductivity of the real water sample, the mixture of 1.0 mL of real water sample and 9.0 mL of HAc‐NaAc solution was used as the electrolyte, and the experiments were performed by standard solution addition for NOR detection due to the extremely low concentration of NOR in Suzhou Creek water (picomole per liter level). With a sensitivity as high as 14.04 µA µM^−1^, which exhibits almost no difference from the optimal condition result (13.81 µA µM^−1^) shown in Figure 3b, even though various interferences such as antibiotics, microplastics, and nitrates are present in the simulated real aqueous environments, indicating considerable application potential. More information is shown in Figures S32 and S33 and Table S5.

Notes: GCE: glass carbon electrode; CPE: carbon paste electrode; LDH: layered double hydroxide; MIP: Molecularly imprinted polymers; CTS: chitosan; EPH: epichlorohydrin; MWCNTs: multi‐walled carbon nanotube; pRGO‐ANSA: the partially reduced graphene oxide‐6‐aminonaphthalene‐2‐sulphonic acid; P‐CDPs: β‐cyclodextrin porous polymers.

### 2.4 Investigation Into the Interaction Mechanism Between Au_1_Pt_1_‐CeO_2_ and NOR

**FIGURE 4:**
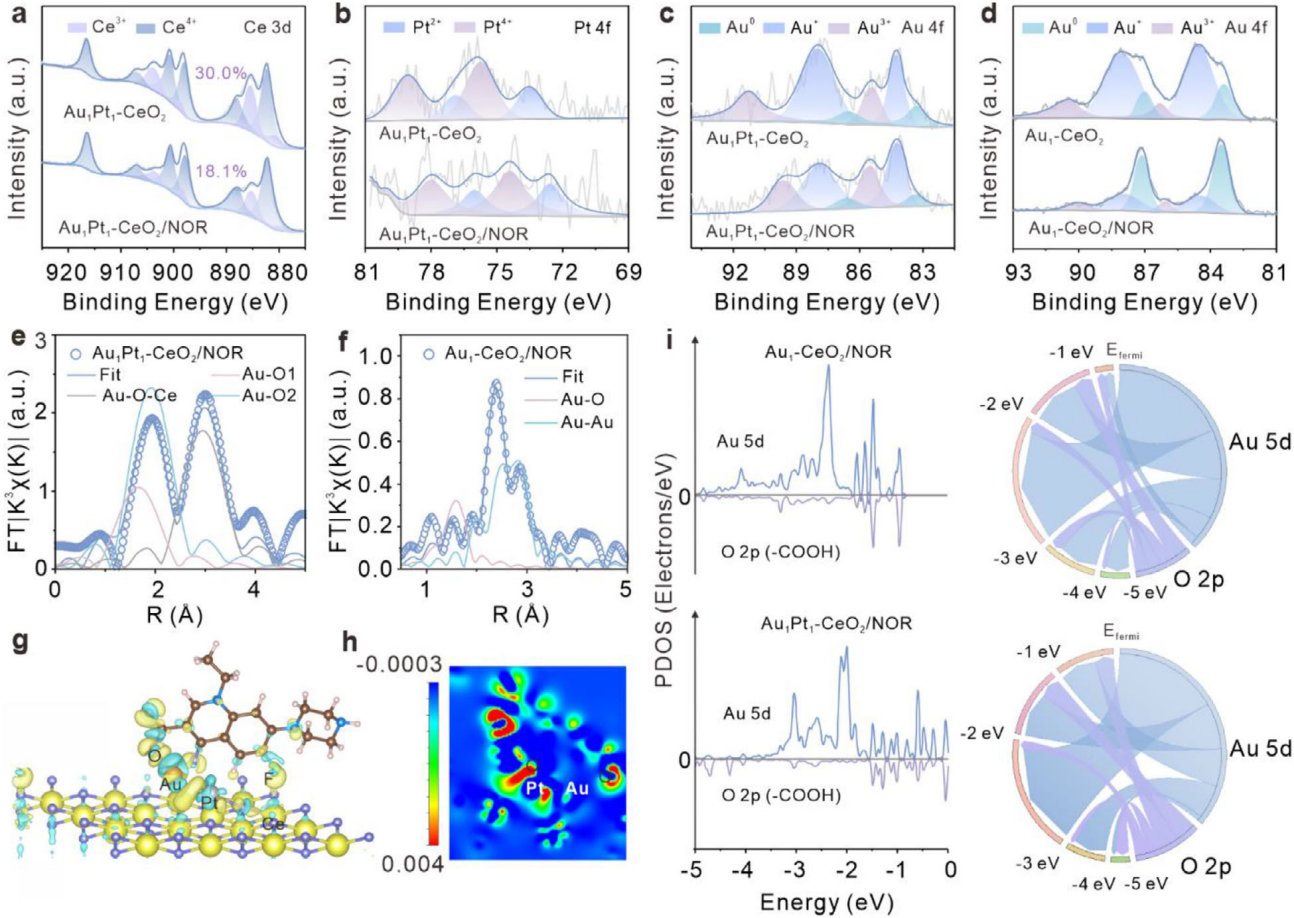
The site recognition between the catalysts and NOR. Comparison of (a) Ce 3d, (b) Pt 4f, (c) Au 4f XPS of Au_1_Pt_1_‐CeO_2_ before and after the adsorption of NOR. (d) Comparison of Au 4f XPS of Au_1_‐CeO_2_ before and after the adsorption of NOR. EXAFS spectra (k^3^‐weighted) of (e) Au_1_Pt_1_‐CeO_2_/NOR and (f) Au_1_‐CeO_2_/NOR in R‐space at the Au L_3_‐edge. (g) Differential charge density image of Au_1_Pt_1_‐CeO_2_/NOR, showcasing the charge transfer between atoms in Au_1_Pt_1_‐CeO_2_ and NOR, is consistent with the XPS analysis results. Blue and yellow represent electron depletion and electron accumulation with the isosurface value of 0.002 e/Bohr^3^, respectively. (h) Differential charge density plot of the cross‐sectional through the Au and Pt atomic layer on Au_1_Pt_1_‐CeO_2_/NOR. The blue area represents electron depletion, and the red area represents electron. (i) Comparison of PDOS (left) for Au 5d and O 2p orbitals in Au_1_‐CeO_2_ and Au_1_Pt_1_‐CeO_2_, where O orbitals are mirrored for clarity of comparison, and the corresponding chord diagrams showing the distribution of electronic orbitals across different energy ranges (right). The chords connecting the orbitals to the energy intervals represent the integrated PDOS (in states), with the width of the chord being proportional to the contribution strength.

The mechanism underlying the superior electrochemical oxidation of NOR over Au_1_Pt_1_‐CeO_2_ was deeply investigated by XPS, XAFS, and DFT calculations. Following the adsorption of NOR, Ce undergoes outward electron transfer with a concomitant decrease in Ce^3+^ content. The characteristic peaks of Au and Pt in the XPS spectra primarily shift to lower energy levels, evidencing interfacial electronic restructuring (Figure 4a–d; Figures S34 and S35). In general, the electron‐loss capacity can be used to directly evaluate the reduction ability of the catalysts, while also indicating a certain interaction between Au and Pt that promotes the charge transfer of Ce and enhances its catalytic reduction ability [64]. Normalized Au L_3_‐edge XANES spectra show that the electron gain of Au after NOR adsorption is less pronounced in Au_1_Pt_1_‐CeO_2_ than in Au_1_‐CeO_2_, indicating that Pt stabilizes the oxidation state of Au while simultaneously weakening charge accumulation at the Au‐O‐Ce interface, thereby promoting charge transfer to NOR (Figure S36). XPS spectra of F 1s indicate that the C‐F peak on the original pyridine ring splits into two peaks after adsorption, and the newly emerged adsorbed state of M‐F peak (located at around 684.00 eV) exhibits the largest integration area in Au_1_Pt_1_‐CeO_2_/NOR (Figure S37) [65]. Combined with the observed Ce^3+^ depletion, it can be inferred that, due to the strong electronegativity of F, charge transfer from Ce to F has occurred. The EXAFS characterization provides further insights into the bonding configuration (Figure 4e,f; Figure S38). In Au_1_Pt_1_‐CeO_2,_ the increase in the coordination number of Au‐O is mainly attributed to its adsorption bonding with O in NOR, which leads to the stretching of Au with O in V‐CeO_2_ (Table S6). Interestingly, Au_1_‐CeO_2_ shows both Au‐O and Au‐Au paths, indicating agglomeration of Au SAs after adsorption reaction, which further corroborates that Pt enhances catalytic stability and structural robustness of Au_1_Pt_1_‐CeO_2_.

The DFT calculation proposed the adsorption configurations of NOR on V‐CeO_2_ and Au_1_Pt_1_‐CeO_2_ (Figures S39 and S40). On V‐CeO_2_, NOR primarily adsorbs at Ov sites, whereas the introduction of AuPt DAs induces a transition toward metal‐site adsorption (Figure S41). These results indicate that V‐CeO_2_ provides an effective adsorption platform, and it is precisely synergistic interactions between Au and Pt optimize the adsorption of NOR at the metal sites, significantly enhancing its electrocatalytic performance. Specifically, Ce preferentially transfers electrons to the highly electronegative F atom, Au interacts with the carboxyl oxygen (‐COOH), and Pt binds mainly with the carbonyl oxygen (‐C = O). An alternative configuration involves Pt interacting with the amine group, though this pathway is less dominant (Figure S42). Differential charge density image and its cross‐sectional image show electron flow from Au to the carboxyl oxygen primarily and from Pt to the carbonyl oxygen, accompanied by charge redistribution around Au sites (Figure 4g,h). Bader charge analysis revealed that Ce acts as the principal electron donor, with Au receiving only 0.05 |e| (Figure S43). Combined with Figure 4d, Pt can weaken the charge buffer effect at the Au‐O‐Ce interface. Concurrently, Ce electron donation behavior toward F highlights the multi‐electron synergistic catalysis in Au_1_Pt_1_‐CeO_2_, which contributes to its strong interference resistance. Figure 4i and Figure S44 indicate the *d*‐band centers of Au and O (‐COOH) in Au_1_Pt_1_‐CeO_2_/NOR approach the Fermi level with aligned peaks compared to Au_1_‐CeO_2_/NOR, indicating that Pt facilitates the activation of Au toward NOR via Au‐O bonding. All of the above demonstrate perfect synergy between Au and Pt atoms. Spatially defined multi‐center interactions account for the high sensitivity and precise recognition of functional groups by Au_1_Pt_1_‐CeO_2_ toward NOR.

### 2.5 Mechanistic Insights Into Stabilization Effect and Catalysis for NOR

**FIGURE 5:**
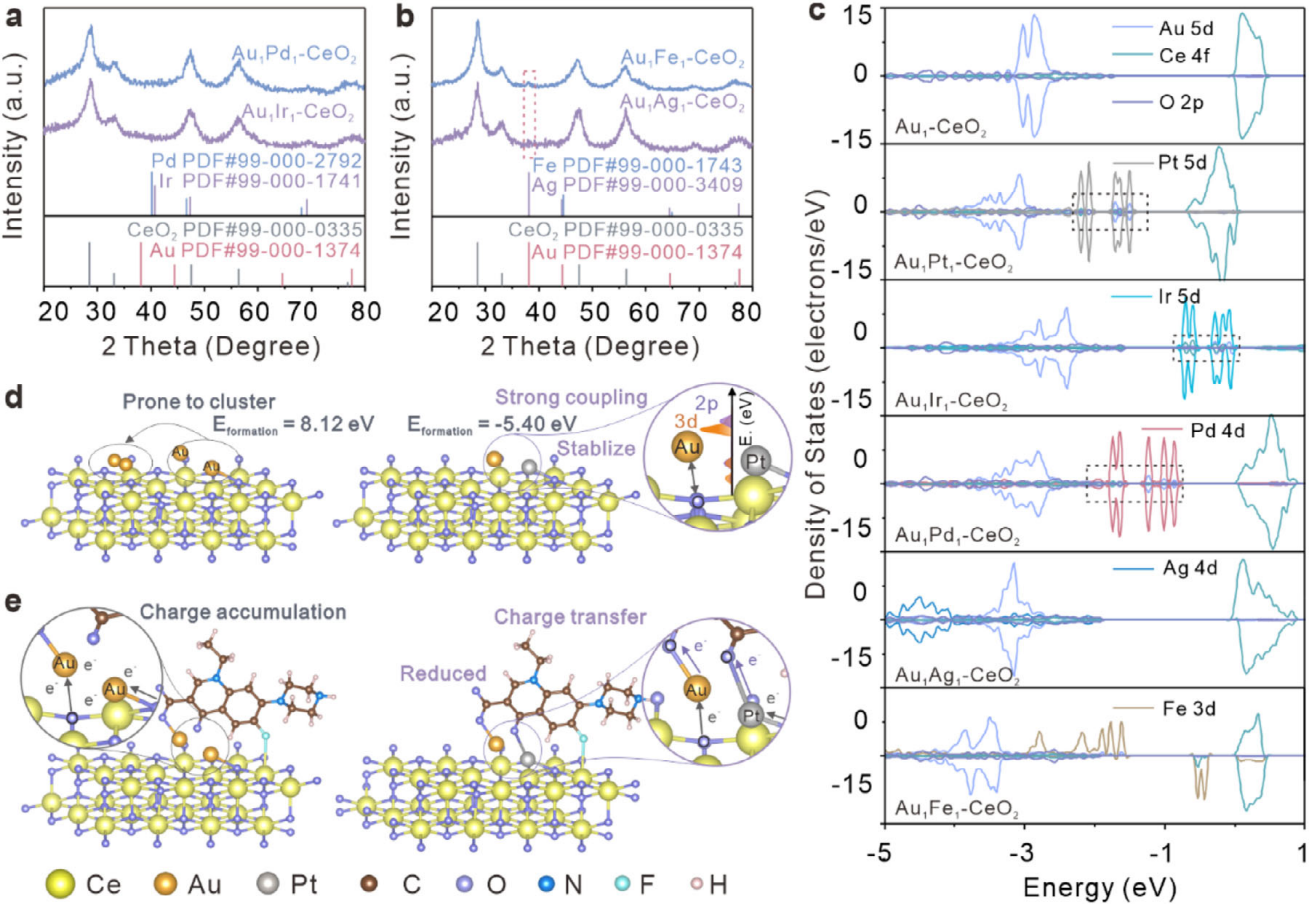
Investigation into the stability mechanism of PGMs (PGMs = Pt, Ir, Pd) on Au and the catalytic mechanism of Au_1_Pt_1_‐CeO_2_ for NOR. XRD spectra of (a) Au_1_Pd_1_‐CeO_2_, Au_1_Ir_1_‐CeO_2_, in which Pd, Ir belong to the platinum group of elements, and (b) Au_1_Fe_1_‐CeO_2_, Au_1_Ag_1_‐CeO_2_, which exhibits distinct Au metallic peaks. (c) PDOS comparison of Au_1_‐CeO_2_, Au_1_Pt_1_‐CeO_2_, Au_1_Ir_1_‐CeO_2_, Au_1_Pd_1_‐CeO_2_, Au_1_Ag_1_‐CeO_2_, and Au_1_Fe_1_‐CeO_2_, where Au_1_PGM_1_‐CeO_2_ displays more well‐matched in Au‐O electron orbital coupling. (d) Mechanism of stabilizing Au SAs on CeO_2_ by Pt enhancement of Au 5d and O 2p orbital coupling. (e) Mechanism illustrating Pt‐enhanced NOR reduction facilitated by interfacial charge transfer at the Au‐O‐Ce active site.

To verify whether the stabilization of Au atoms can be generalized beyond Pt, we extended the study to other transition metal elements. Given the critical role of structural stability in catalytic performance, Au‐platinum‐group metals (PGMs = Pd, Ir), alongside selected non‐PGM elements (Ag, Fe), were synthesized for comparison to systematically evaluate their ability to stabilize Au and form robust heteronuclear diatomic structures. The stabilization effect was confirmed through experimental analysis and DFT calculations. In XRD patterns of Au_1_Fe_1_‐CeO_2_ and Au_1_Ag_1_‐CeO_2_ shown in Figure 5b, the metal peaks of Au are very pronounced, whereas the metal peaks of Au are almost absent in the Au‐PGM DACs (Figure 5a). XPS analysis further confirms that, compared to Ag and Fe, Pd and Ir can effectively moderate the reduction degree of CeO_2_, providing more stable anchoring sites for Au atoms (Figures S45–S47).

DFT calculations were performed to determine the optimized configurations and formation energies of AuM‐CeO_2_ (M = Ir, Pd, Ag, Fe), as shown in Figure S48, revealing that the Au‐PGM systems exhibit lower formation energies (−0.45 and 0.10 eV), in contrast to Au‐nonPGM systems (3.34 and 2.78 eV). The impact of PGMs on the interaction between Au and CeO_2_ was further elucidated through PDOS analysis. As shown in Figure 5c, the comparison of the PDOS for Au_1_‐CeO_2_ before and after introducing Ag, Fe, Pd, and Ir demonstrates that in the Au‐PGM diatomic models, the electronic states of Au undergo significant redistribution, giving rise to new peaks that exhibit a high degree of matching with the O 2p states (highlighted by dashed boxes). This indicates enhanced electron coupling between Au and O after PGM introduction. Comprehensive observations of Au_1_Ag_1_‐CeO_2_ and Au_1_Fe_1_‐CeO_2_ reveal minimal changes in Au 5d and O 2p states, with no evident overlap at comparable energy levels. PGMs effectively strengthen the interaction between Au and O atoms in CeO_2_, increasing the covalent character of Au‐O bonds, stabilizing Au within more robust diatomic structures. Based on the above electronic structure analysis, the stabilization mechanism of Au by Pt is schematically illustrated in Figure 5d, and the synergistic effect within this stable AuPt configuration was found to be crucial for the NOR catalysis, as depicted in the proposed reaction mechanism in Figure 5e, underscoring the dual role of Pt in simultaneously achieving structural stabilization and catalytic activation.

## 3 Conclusions

In this work, we have unveiled a generalizable adjacency‐assisted strategy that concurrently addresses the twin challenges of instability and monofunctionality in Au single‐atom catalysis. We achieved Pt‐induced stabilization of Au atoms through enhanced Au‐O orbital coupling, while simultaneously creating cooperative multi‐center sites for the spatially precise recognition of SCMMs. This dual functionality enables the Au_1_Pt_1_‐CeO_2_ catalyst to exhibit exceptional sensitivity, selectivity, and robustness in the electrochemical catalysis of NOR, even in complex real‐world aqueous environments. The Pt‐induced stabilization effect observed in Au_1_Pt_1_‐CeO_2_ can be extended to other PGMs (Ir and Pd), which provide analogous electronic modulation. In contrast, elements like Ag and Fe, lacking partially filled d‐orbitals with suitable energy alignment for effective charge redistribution, fail to facilitate comparable stabilization. This work establishes a strategy for multicenter active‐site catalysts toward SCMMs, providing a clear criterion for the rational design of stable catalytic systems.

## Funding

The National Key R&D Program of China (2023YFE0122500), the National Natural Science Foundation of China (42407608), the Dawn Scholar Program of Shanghai Education Commission (No. 23SG37), and China Postdoctoral Science Foundation Project (2024M751938). The authors thank the staff of the BL17B beam lines at the Shanghai Synchrotron Radiation Facility (SSRF) for data collection.

## Conflicts of Interest

The authors declare no conflicts of interest.

## Supporting information




**Supporting File**: advs74720‐sup‐0001‐SuppMat.docx.

## Data Availability

The data that support the findings of this study are available from the corresponding authors upon reasonable request.
