# Supplementary material for: Stabilizing Atomically Dispersed Au With Adjacent Pt for Spatially Precise Molecule Recognition
Source: Adv Sci (Weinh). 2026 Mar 9;13(28):e74720. doi: 10.1002/advs.74720 (PMC13185823; doi:10.1002/advs.74720)
Supplement: Supplementary file 1 — Supporting File: advs74720‐sup‐0001‐SuppMat.docx. [file ADVS-13-e74720-s001.docx]

Supporting Information

Stabilizing Atomically Dispersed Au with Adjacent Pt for Spatially Precise Molecule Recognition

Rui Tang, ^‡^ Xiangyu Xiao, ^‡,^ * Jingyi Yao, Qing Wang, Qijing Gao, Nan Zhou, Fanyi Kong, Dingguan Liang, Kangning Dong, Liang Xiong, Yuwei Cao,* Zhendong Lei,* and Liang Tang*

*Corresponding author

Email addresses: xxy69@tongji.edu.cn;

caoyuwei9@gmail.com;

leizd95@tongji.edu.cn;

tang1liang@shu.edu.cn.

Author Contributions

R. T. and X. X. contributed equally.

**1. Experimental Section**

**1.1. Materials**

Chloroplatinic acid hexahydrate [H_2_PtCl_6_•6H_2_O], hydrogen tetrachloroaurate hydrate [HAuCl_4_•4H_2_O], silver nitrate standard solution (AgNO_3_), Ferric nitrate nonahydrate (Fe(NO)_3_•9H_2_O), and ammonium carbonate [(NH_4_)_2_CO_3_] were purchased from Aladdin Industrial Corporation (Shanghai, China)**.** dihydrogen hexachloroiridate (IV) xhydrate [H_2_IrCl_6_•xH_2_O] was purchased from Bide Pharmaceutical Technology Co., Ltd. (Shanghai, China). Palladium (II) chloride (PdCl_2_) was purchased from Haohong Biopharmaceutical Technology Co., Ltd. (Shanghai, China). Ethylene glycol and cerium nitrate hexahydrate [Ce(NO_3_)_3_•6H_2_O] were purchased from Sinopharm Chemical Reagent Co., Ltd. (Shanghai, China). Polyvinylpyrrolidone (PVP Mw≈40000) was purchased from Titan (Shanghai, China). Norfloxacin (NOR) was obtained from Tokyo Chemical Industry Co., Ltd (Shanghai, China). All aqueous solutions were prepared using deionized water with a resistivity of 18.2 MΩ cm^-1^.

**1.2. Characterizations**

The X-ray diffraction (XRD) was performed on a Bruker diffractometer (D8 advance, Cu Kα radiation λ = 1.54086 Å) equipped with a Lynxeyes detector. The morphology and atom distribution of the catalysts were characterized by SEM (ZEISS Gemini 300) and high-angle annular dark-field scanning transmission electron microscopy (HAADF-STEM, JEOL ARM-200F) with a spherical aberration (Ac-STEM). The X-ray photoelectron spectroscopy (XPS) measurements were operated on Thermo Scientific K-Alpha spectrometer with an Al Kα X-ray source (1486.6eV). The pair distribution function (PDF) experiments were performed at the BL17B1 beamline of the National Protein Science Facility at the Shanghai Synchrotron Radiation Facility (SSRF), using an X-ray energy of 17.5 keV (λ=0.7085 Å). All samples were sealed with kapton films and diffraction data were collected with a two-dimensional X-ray area detector in transmission geometry. The results were Fourier transformed using PDFgetX3 initially, then refined and fitted by PDFgui.^[1,2]^ The contents of Au and Pt in the catalysts were quantified using inductively coupled plasma optical emission spectrometry (ICP-OES, Agilent ICP-5110 OES, USA). All electrochemical experiments were conducted by a CHI760 computer-controlled potentiostat (ChenHua Instruments Co., Shanghai, China).

**1.3. Preparation of CeO_2_ nanospheres with oxygen-rich vacancy**

The synthesis of CeO_2_ referred to the previous literature. A total of 0.5 g Ce(NO_3_)_3_·6H_2_O and 0.2 g PVP were dissolved in 15 mL of ethylene glycol. To this solution, 1 mL of deionized water was added slowly. After stirring continuously for 30 minutes, the resulting clear solution was transferred to a 25 mL Teflon-lined autoclave and heated at 160 °C for 8 hours. The product, cooled to room temperature, was washed several times with DI water and ethanol and dried at 60 °C overnight.

**1.4. Preparation of SACs and Au-based bimetallic catalysts**

V-CeO_2_ supported Au-M (M = Pt, Ir, Pd, Ag, Fe) bimetallic single atom by deposition-precipitation method.^[3]^ Typically, 0.2 g of V-CeO_2_ is dispersed in deionized water for 15 min. Then, (NH_4_)_2_CO_3_ was added to alkalize the suspension. 4 mL of H_2_PtCl_6_•6H_2_O, AgNO_3_, Fe(NO)_3_•9H_2_O, H_2_IrCl_6_•xH_2_O, and PdCl_2_ solution at 1 mg mL^-1^ each are added dropwise simultaneously with HAuCl_4_•4H_2_O (1 mg mL^-1^) into V-CeO_2_ suspension, while stirring at 500 rpm for 1 h and following aging at room temperature for 1 h. After several centrifugal washes with deionized water, the product was freeze-dried overnight to obtain Au_1_M_1_-CeO_2_. Preparation of AuPt alloy-CeO_2_ involves rapidly pouring the precursors during addition and hot drying at 60 ℃. The preparation process of Au_1_-CeO_2_ and Pt_1_-CeO_2_ was the same as that of Au_1_M_1_-CeO_2_, with the only difference being that only one metal precursor was added.

**1.5. Standard Solutions and Supporting Electrolyte**

1 mM norfloxacin stock solution was prepared by taking a certain amount of norfloxacin powder and adding an appropriate amount of deionized water with dilute hydrochloric acid as cosolvent. Norfloxacin solutions with different target concentrations were diluted with deionized water and stored at a low temperature. HAc-NaAc buffer solution was prepared by mixing 0.1 M sodium acetate (NaAc) and 0.1 M acetic acid (HAc) in the appropriate proportions to achieve the desired pH.

**1.6. Fabrication of Modified Electrodes**

The prepared samples (1 mg) were uniformly dispersed into 1 mL deionized water to obtain 1 mg mL^-1^ of sample suspensions. The glass carbon electrode (GCE, ChenHua Instruments Co., Shanghai, China) polished by alumina powder was ultrasound with nitric acid solution (volume ratio 1:1), anhydrous ethanol, and deionized water. Take the above sample suspensions and drop 10 μL on the surface of GCE. The modified electrodes were dried naturally at room temperature for 12 h.

**1.7. Adsorption Experiments**

10 mg of V-CeO_2_, Au_1_-CeO_2_, Pt_1_-CeO_2_, and Au_1_Pt_1_-CeO_2_ were dispersed in 10 mL (0.1 M, pH 6.0) of HAc-NaAc electrolyte containing 100 μM NOR, respectively. The suspension was then oscillated on a shaker at 288 K for 24 h. After full adsorption, the samples were washed several times by centrifugation with HAc-NaAc buffer solution to remove additional NOR. Finally, they were freeze-dried for 12 h to obtain the adsorbed solid powder.

**1.8. Sensitivity Calculation**

Sensitivity was obtained from the slope of the linear calibration plot of peak current *I* versus analyte concentration *C* using least-squares linear regression:

$$I=kC+a$$

where *a* is the intercept and the sensitivity was defined as the slope *k*. The slope *k* was calculated as:

$$k=\frac{\sum(x_{i}-\bar{x})(y_{i}-\bar{y})}{\sum(x_{i}-\bar{x})^{2}}$$

where $\bar{x}$ and $\bar{y}$ are the mean values of concentration and peak current, respectively, *i* indexes each data point, and the summations run over all calibration points.

**1.9. XAFS Data Analysis**

The Ce L_3_-edge, Pt L_3_-edge, and Au L_3_-edge XAFS spectra were tested in Lytle-fluorescence mode at the BL17B beamline of Shanghai Synchrotron Radiation Facility (SSRF). Athena software was used to lower the pre- and post-edge background signal and to normalize spectra intensity. Artemis software was used to apply the Fourier transform on the data and fit its contours.

**1.10. Computational Methods**

All of the calculations are performed in the framework of the density functional theory with the projector augmented plane-wave method, as implemented in the Vienna ab initio simulation package (VASP)^[4,5]^. The generalized gradient approximation (GGA) proposed by Perdew, Burke, and Ernzerhof (PBE) is selected for the exchange-correlation potential^[6,7]^. The long-range van der Waals interaction is described by the DFT-D3 approach^[8]^. The cut-off energy for plane wave is set to 480 eV. The energy criterion is set to 10^−5^ eV in iterative solution of the Kohn-Sham equation. All the structures are relaxed until the residual forces on the atoms have declined to less than 0.01 eV/Å. Data analysis and visualization are carried out with the help of VASPKIT^[9]^ code and VESTA^[10]^. To avoid interlaminar interactions, a vacuum spacing of 20 Å is applied perpendicular to the slab.

The adsorption energy E_ads_ is expressed as

|  | $\Delta E_{ads}=E_{A+B}-E_{A}-E_{B}$ |
| --- | --- |

where $E_{A+B}$ is the total energy of slab A model with B adsorption, $E_{A}$ is the energy of a A slab, and $E_{B}$ is that for a B molecule.

Here, we define $\Delta\rho=\rho_{A+B}-\rho_{A}-\rho_{B}$ as the differential charge density of A/B heterostructure, where $\rho_{AB}$, $\rho_{A}$ and $\rho_{B}$ are the charge densities of A/B heterostructure, isolated A and B slabs, respectively.

We use the Bader charge to express the charge transfer quantity.

The formation energy E_form_ is expressed as

$$E_{form}=E_{defect}-E_{perfect}-{\sum E}_{vacancy}$$

where $E_{defect}$ and $E_{perfect}$ are the energy with and without the Au and Pt atoms. $E_{vacancy}$ are the energy of the vacancy atom, respectively.

**2. Figures**


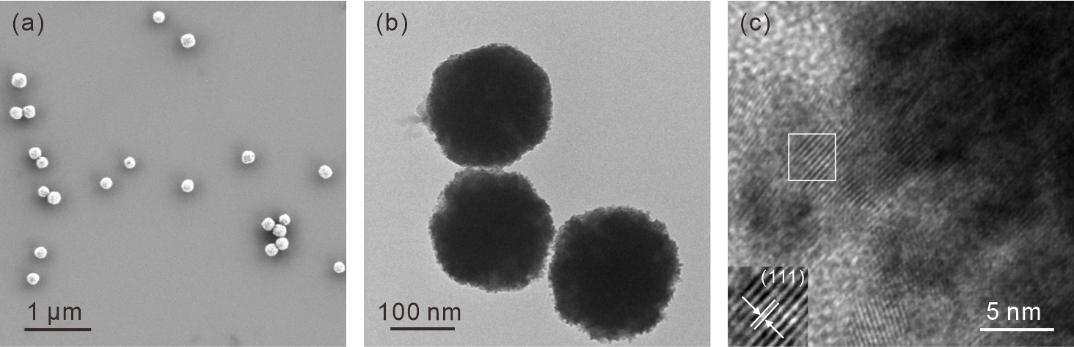


**Figure S1.** (a) SEM and (b, c) TEM images of V-CeO_2_. The insert in Figure S1c showed an enlarged TEM image of the corresponding selected area.

The synthesized V-CeO_2_, as seen in Figure S1, exhibits a typical spherical morphology with a diameter of about 150 nm, indicating the formation of well-defined nanosphere structures. The particles are uniform in size and have rough surfaces, with the crystal surface (111) outlined in the white box of Figure S1c, confirming the successful synthesis of V-CeO_2_.


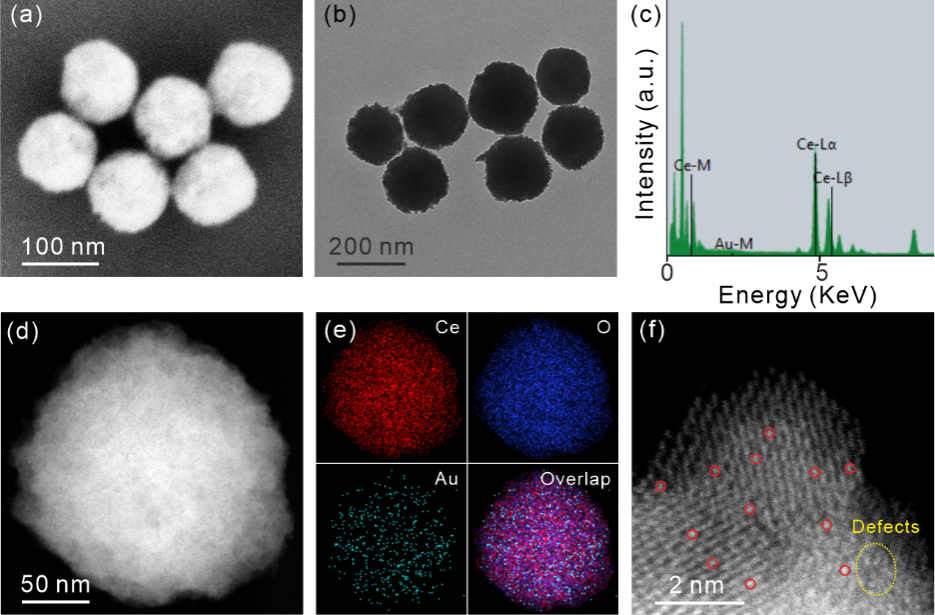


**Figure S2.** (a, b, c) SEM, TEM images, and EDS of Au_1_-CeO_2_. (d, f) HAADF-STEM images of Au_1_-CeO_2_. Au single atoms were circled in red. Yellow dashed lines circled the defects in the V-CeO_2_ substrate. (e) Elemental mapping of Ce, O, Au, and their overlap corresponding to (d).

EDS analysis shows distinct small peaks at about 2.12 keV (Au M), confirming the successful incorporation of the noble metal (Figure S2c). The Au atoms exhibit a uniform dispersion pattern on the V-CeO_2_ carrier with no observable localized regions of enrichment in Figure S2e. Surface scanning analysis quantitatively confirms the uniform distribution, showing consistent metal signal intensity over the entire scanning area. Figure S2f indicates that the Au atoms were atomically dispersed (bright white dots circled by red).


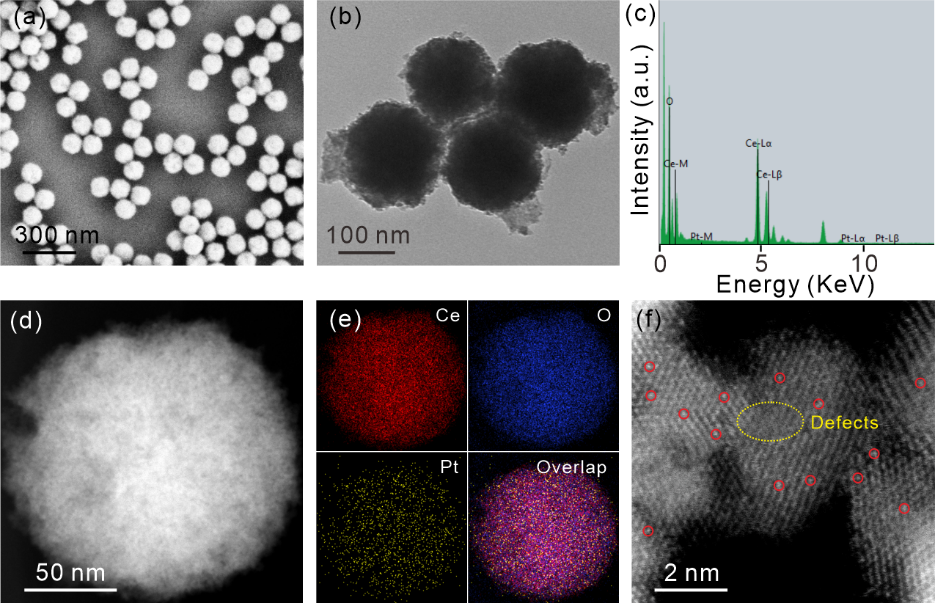


**Figure S3.** (a, b, c) SEM, TEM images, and EDS of Pt_1_-CeO_2_. (d, f) HAADF-STEM images of Pt_1_-CeO_2_. Pt single atoms were circled in red. Yellow dashed lines circled the defects in the V-CeO_2_ substrate. (e) Elemental mapping of Ce, O, Pt, and their overlap image corresponding to (d).

EDS analysis (Figure S3c) shows a tiny peak at about 2.05 keV (Pt M), corroborating that Pt atoms are loaded and there were no other hetero-elements. The elemental mapping image in Figure S3e confirms that the Ce, O, and Pt atoms are uniformly distributed, with no aggregation observed, as highlighted by the bright white dots encircled in red. Figure S3f demonstrates the atomic dispersion of Pt atoms.


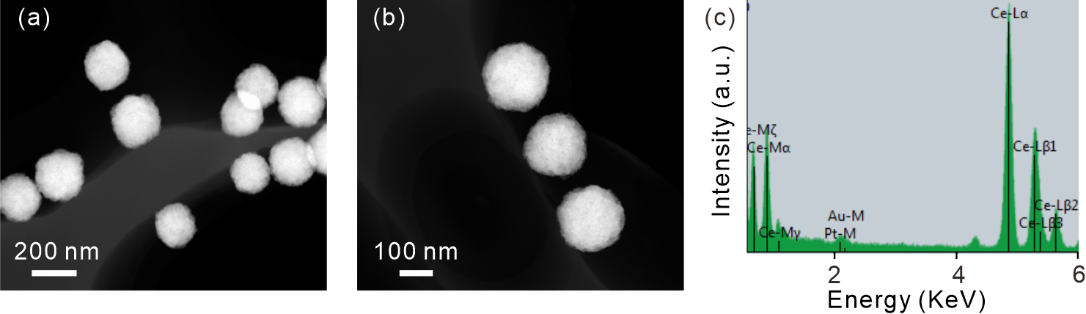


**Figure S4.** (a, b) HAADF-STEM images of Au_1_Pt_1_-CeO_2_ at different magnifications. (c) EDS of Au_1_Pt_1_-CeO_2_.

*
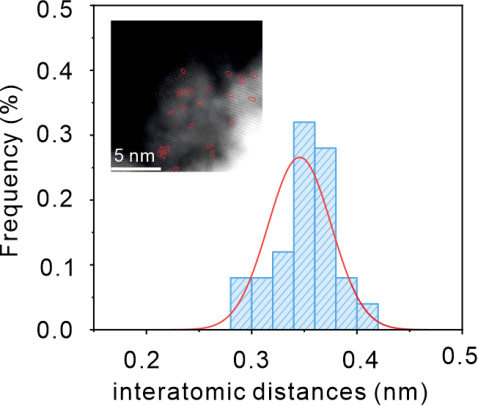
*

**Figure S5.** HAADF-STEM of Au_1_Pt_1_-CeO_2_ (inset) and the distance distribution histogram of Au and Pt atoms on CeO_2_.


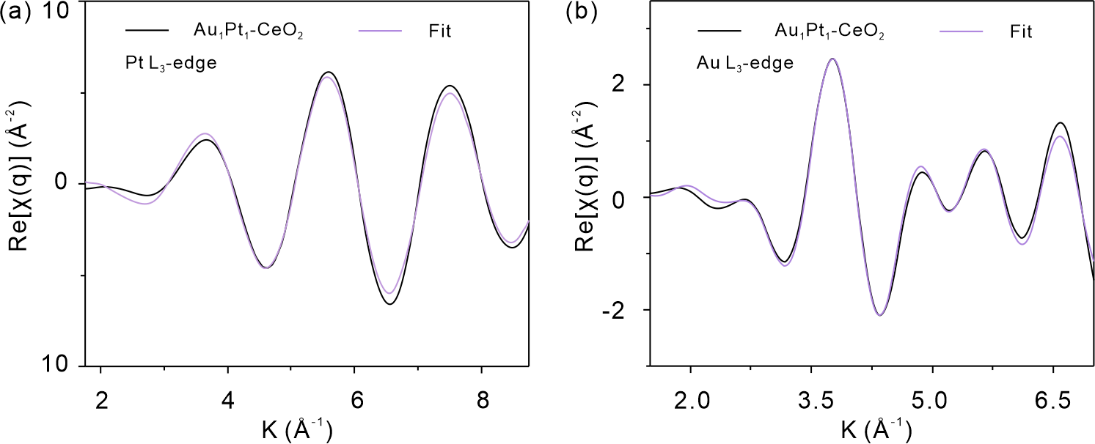


**Figure S6.** The corresponding k^3^χ(k) oscillations of Au_1_Pt_1_-CeO_2_ in Figures 1h-i.


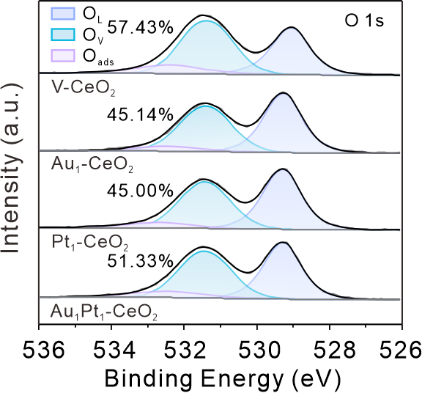


**Figure S7.** XPS of O 1s in V-CeO_2_, Au_1_-CeO_2_, Pt_1_-CeO_2_, and Au_1_Pt_1_-CeO_2_; the numbers in the figure represent the percentage amount of O_V_.


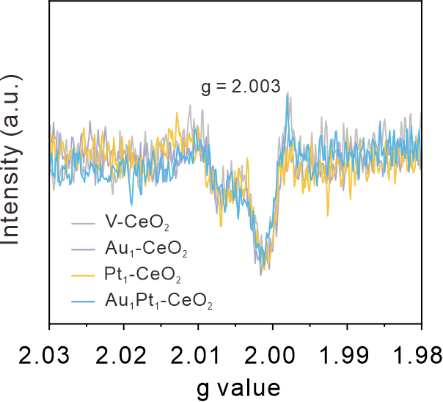


**Figure S8**. ESR spectra of V-CeO_2_, Au_1_-CeO_2_, Pt_1_-CeO_2_ and Au_1_Pt_1_-CeO_2_.


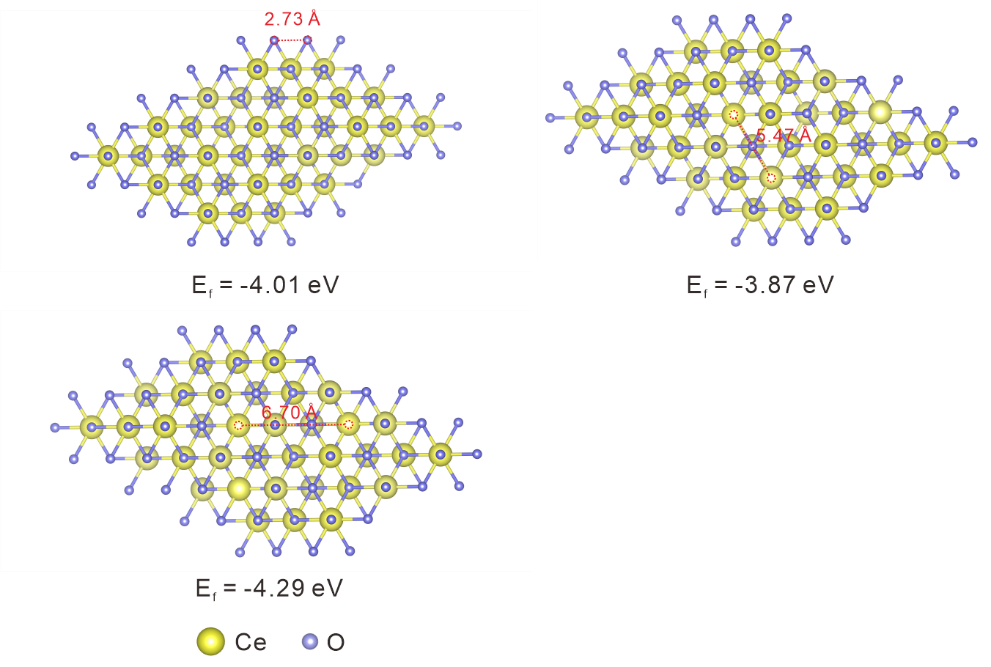


**Figure S9**. The configurations and corresponding formation energies of V-CeO_2_ with different vacancy distances.

To evaluate the effect of vacancy position on the vacancy formation energetics of CeO_2_, three representative Ov configurations were constructed on the same CeO_2_ surface model. In each configuration, two lattice oxygen atoms were removed to generate two Ov sites, while varying the relative spatial separation between the two vacancy sites. Figure S9 shows the top-view structures of these three configurations, where vacancy sites (unoccupied lattice oxygen positions) are marked by red circles and corresponding separations are labeled as (d_Ov-Ov_ = 2.73, 5.47, and 6.70 Å), representing states where the two vacancies in relatively close proximity, at an intermediate distance, and far apart, respectively. Although the calculated energy differ among the three configurations are small, the results suggest that the vacancy spacing can influence the energetic stability of Ov on the CeO_2_ surface. This comparison provides theoretical guidance reference for optimizing the arrangement of Ov.


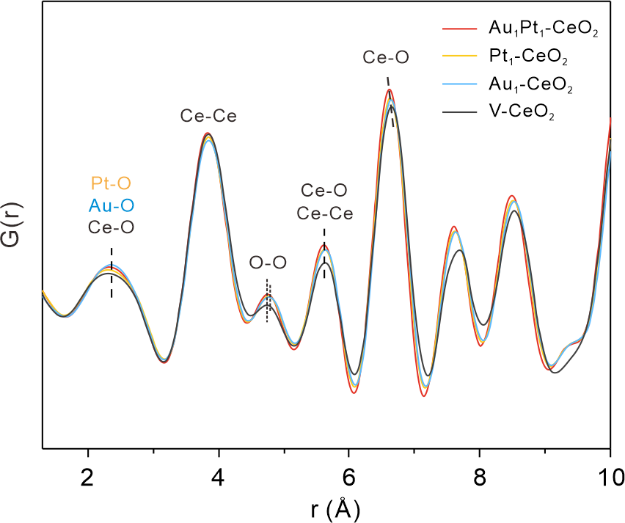


**Figure S10.** PDF refinement fitting of Au_1_Pt_1_-CeO_2_, Au_1_-CeO_2_, Pt_1_-CeO_2_, and V-CeO_2_.

The PDF values of the materials are obtained from Figure S10. At around 2.30 Å, the peak areas of Au-O, Pt-O, and Ce-O increase, indicating that diatomic loading provided more metal-oxygen sites,^[11–13]^ reflecting the enhanced metal-oxygen interaction. For O-O pairs at about 4.70 Å, the monatomic loading may induce local lattice expansion effects leading to the elongation of the O-O distances, whereas the diatomic loading effectively relieves the lattice stress through its synergistic effect, which returns the average bond lengths of the O-Os to a level close to that of the pristine V-CeO_2_, and thus the lattice structure tends to be in equilibrium. On the other hand, the shortening of Ce-O distance at about 6.60 Å is consistent with the XAFS results, indicating that the diatomic loading improves the stability of Au_1_Pt_1_-CeO_2_ effectively.


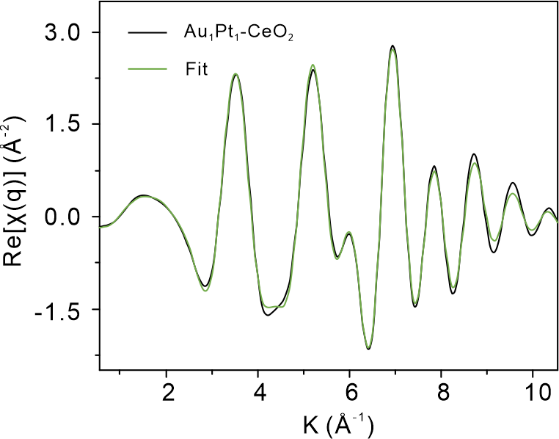


**Figure S11.** The corresponding k^3^χ(k) oscillations of Au_1_Pt_1_-CeO_2_ in Figure 2d.

^
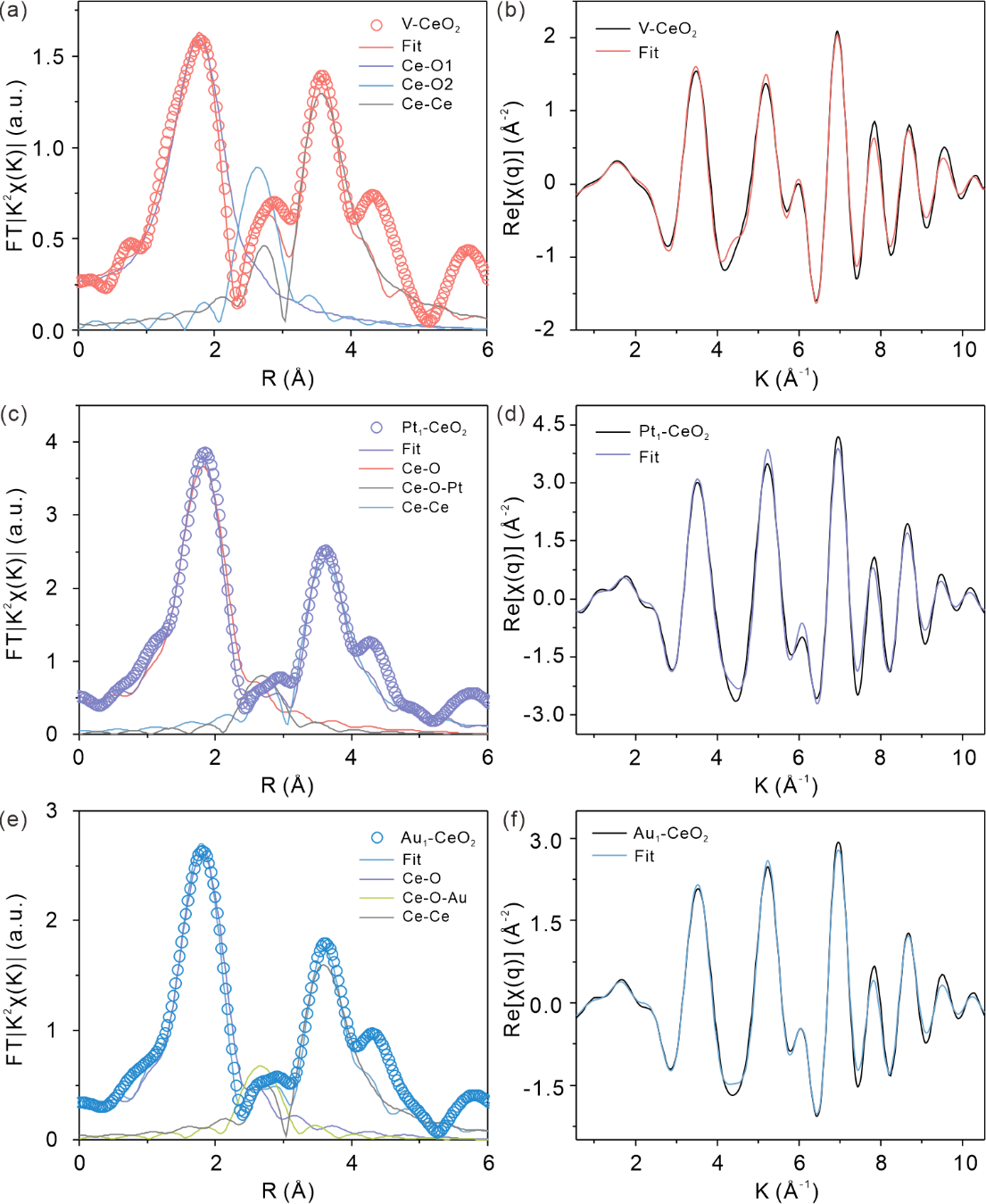
^

**Figure S12.** (a, c, e) The Ce L_3_-edge EXAFS spectra in R space (k^3^-weighted) and the fitting curves of V-CeO_2_, Pt_1_-CeO_2_, and Au_1_-CeO_2_. (b, d, f) The corresponding k^3^χ(k) oscillations of V-CeO_2_, Pt_1_-CeO_2_, and Au_1_-CeO_2_.


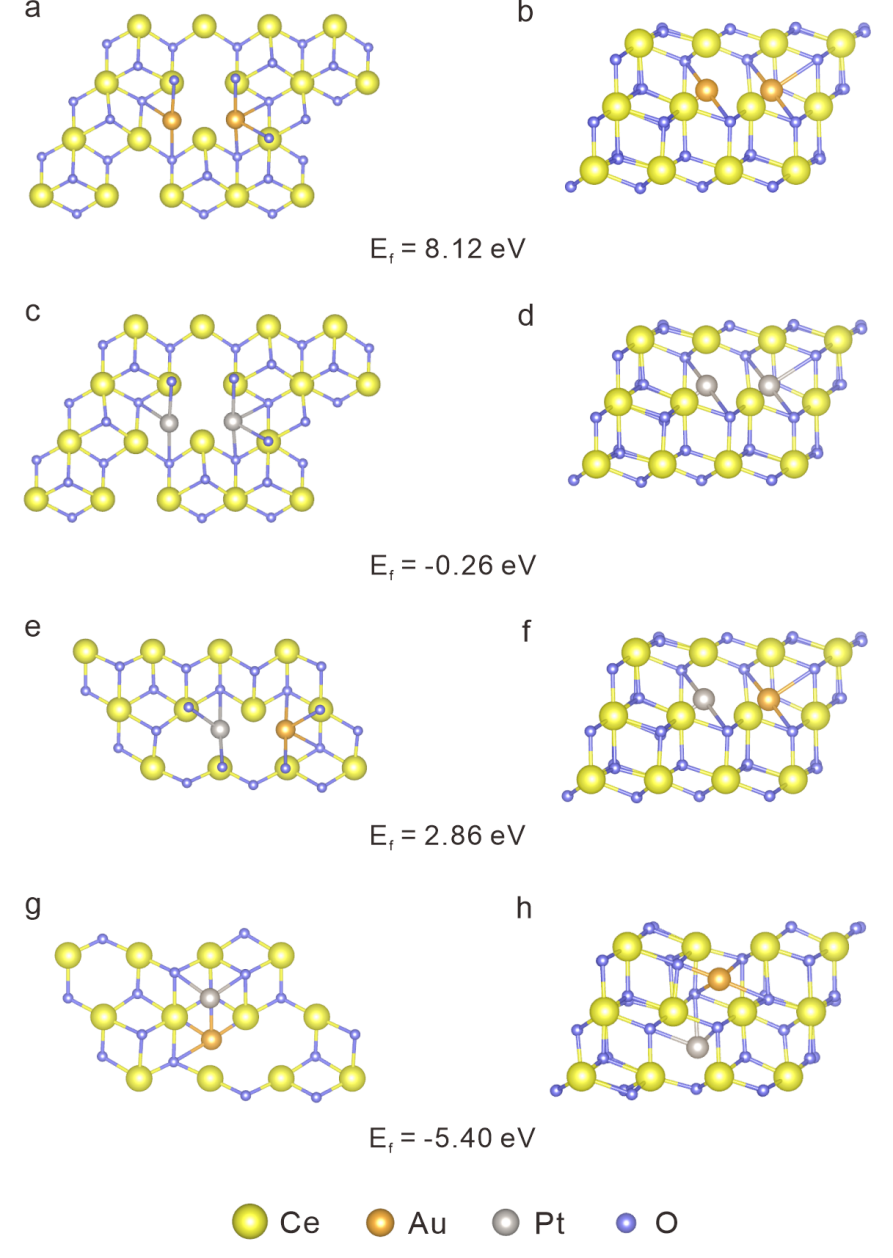


**Figure S13.** The optimized configurations and corresponding formation energy of (a, b) Au-CeO_2_, (c, d) Pt-CeO_2_, (e, f) Au_1_Pt_1_-CeO_2_ with Au, Pt at relative position level, (g, h) Au_1_Pt_1_-CeO_2_ with Au, Pt atoms tilted relative to position and closer to O in side view and top view, respectively.

DFT calculations reveal three structurally optimized Au-CeO_2_, Pt-CeO_2_, and Au_1_Pt_1_-CeO_2_ configurations. The results show that the formation energy (E_f_) is 8.12 eV for Au-CeO_2_ and -0.26 eV for Pt-CeO_2_, indicating that Au isolated on V-CeO_2_ is relatively unstable. Figures S13 (e, f) and (g, h) both show the structure of Au and Pt replacing the position of the original oxygen atom, while their relative positions are different. The E_f_ of the two structures of Au_1_Pt_1_-CeO_2_ were 2.86 eV and -5.40 eV, respectively, suggesting that the configuration in which Au and Pt are relatively tilted and closer to oxygen has the highest thermodynamic stability.


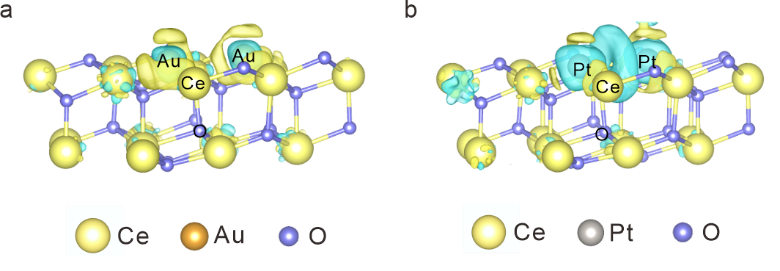


**Figure S14.** Differential charge density images of (a) Au-CeO_2_ and (b) Pt-CeO_2_. The blue areas represent electron depletion, the yellow areas represent electron accumulation.

Figure S14 shows that electron loss occurred around the Au atoms, indicating that Au acts as an electron donor. However, the area of its electron accumulation region seems to be larger, which may imply that there is a redistribution of charge at the interface and that there is a charge feedback mechanism between V-CeO_2_ and Au.

The area of Pt electron loss is larger than the electron accumulation area, indicating that Pt donates electrons in Pt-CeO_2_, and Ce has both electron loss and accumulation, which may suggest that Ce has undergone charge transfer in the Pt-CeO_2_ sample, with some areas losing electrons while the other part obtains electrons. The variation between the Ce^3+^ and Ce^4+^ oxidation states of V-CeO_2_ or the presence of oxygen vacancies allows it to act as a charge adjustment.


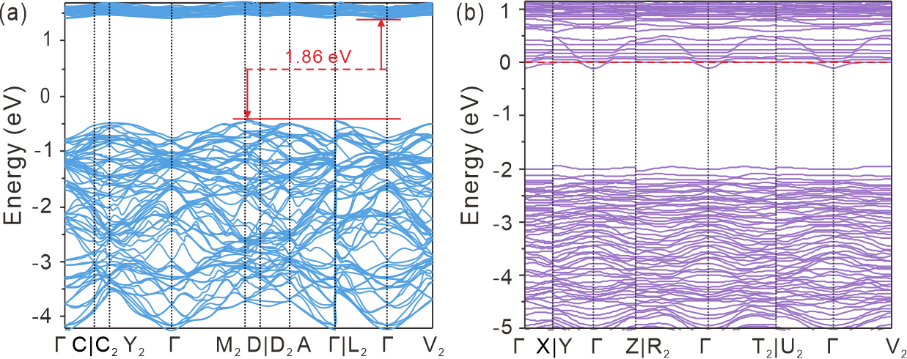


**Figure S15.** Band structure diagrams of (a) CeO_2_, and (b) V-CeO_2_.

As illustrated in Figure S15, the comparative analysis of electronic structures demonstrates that the introduction of oxygen vacancies in CeO_2_ leads to the formation of localized occupied states near the Fermi surface (E_fer_), in contrast to the pristine CeO_2_. This structural modification significantly alters the electronic configuration, resulting in enhanced electrical conductivity through the creation of additional charge transport pathways.


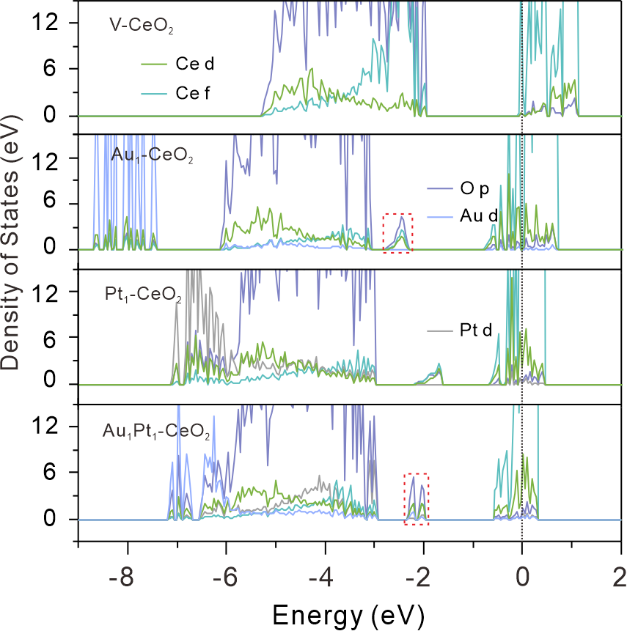


**Figure S16.** Comparison of PDOS diagram of V-CeO_2_, Au_1_-CeO_2_, Pt_1_-CeO_2_, and Au_1_Pt_1_-CeO_2_ exhibits enhanced electronic continuity near the Fermi level in Au_1_Pt_1_-CeO_2_.


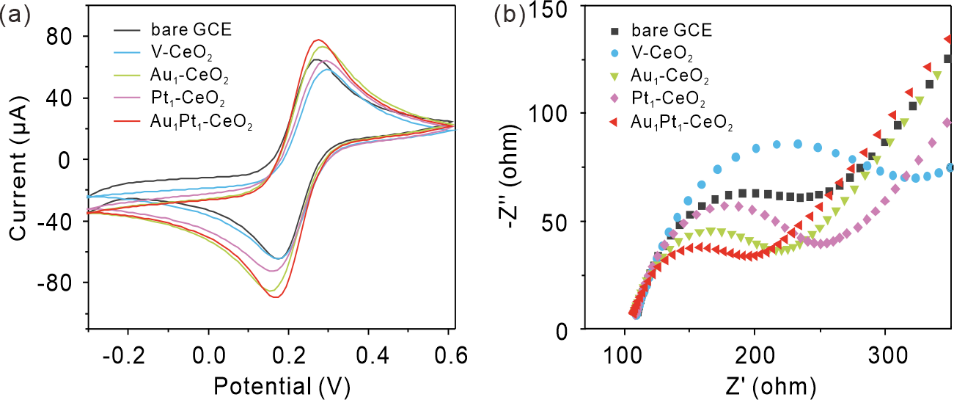


**Figure S17.** (a) CV and (b) EIS corresponding to bare GCE, Au_1_Pt_1_-CeO_2_, Au_1_-CeO_2_, Pt_1_-CeO_2_, and V-CeO_2_, respectively. Experiments were carried out in a 5.0 mM Fe(CN)_6_^3-/4-^ containing 0.1 M KCl.

Fe(CN)_6_^3-/4-^ solution is used as an ion probe to investigate the charge transport properties of the materials. From Figure S17a, V-CeO_2_ has the worst conductivity due to the lowest redox peak current value. Compared with Au_1_-CeO_2_ and Pt_1_-CeO_2_, Au_1_Pt_1_-CeO_2_ has the highest current value, indicating that there is a certain interaction between Au and Pt single atoms that can promote the charge transfer rate. From Figure S17b, the semi-circle of the EIS curve of Au_1_Pt_1_-CeO_2_ modified electrode indicates that the electron-transfer resistance (R_et_) is 42 Ω, which is lower than that of other modified materials (the R_et_ of Au_1_-CeO_2_, Pt_1_-CeO_2_, V-CeO_2_, and bare GCE are about 55 Ω, 71 Ω, 100 Ω, and 63 Ω, respectively), further demonstrating that the loading of the Au-Pt diatoms can promote the electrochemical activity effectively.

.


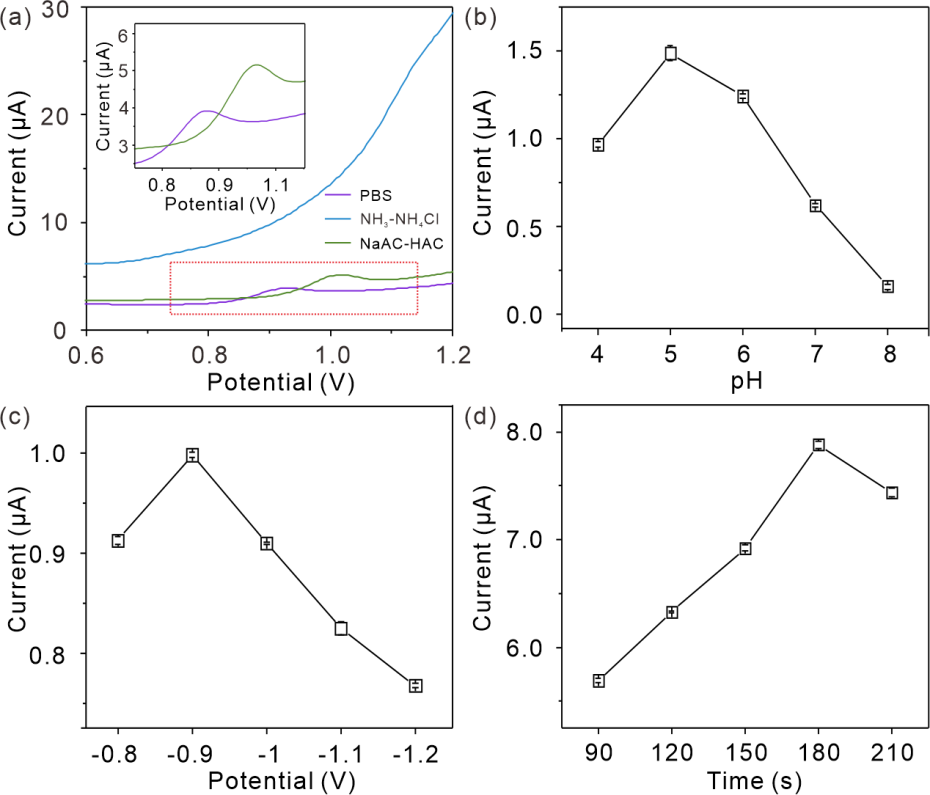


**Figure S18.** Parameter optimization for electrochemical catalysis of NOR with Au_1_Pt_1_-CeO_2_.

Optimization of experimental conditions was carried out first. As shown in Figure S18a, the HAc-NaAc buffer condition of norfloxacin has the most pronounced current response compared to PBS buffer and NH_3_-NH_4_Cl buffer. This may be due to its low pH, which favors the protonated state of NOR and improves the interaction with the electrode material. When pH=5, the electrochemical activity of NOR may be at its optimum, and its reaction rate with the electrode surface is higher. At lower pH, the high concentration of H^+^ will be too high, which may lead to the formation of too much H_2_ or other side reactions on the electrode surface, reducing the electrochemical activity. When the pH value is too high, the NOR molecules will be more in the deprotonated state, weakening their adsorption on the electrode surface, hence decreasing the current response.

As shown in Figure S18c, the enrichment efficiency of norfloxacin is maximum at an enrichment voltage of -0.90 V. Too low of enrichment voltage may result in insufficient adsorption of NOR molecules, while too high of enrichment voltage will cause the occurrence of side-reactions or the desorption of NOR molecules, which can reduce the signal intensity. Longer enrichment time usually improves the binding strength of NOR molecules to the electrode modification material, while over-enrichment may be diffusion-limited, thereby leading to desorption of NOR molecules or adsorption of impurity molecules, which reduces the current accordingly.


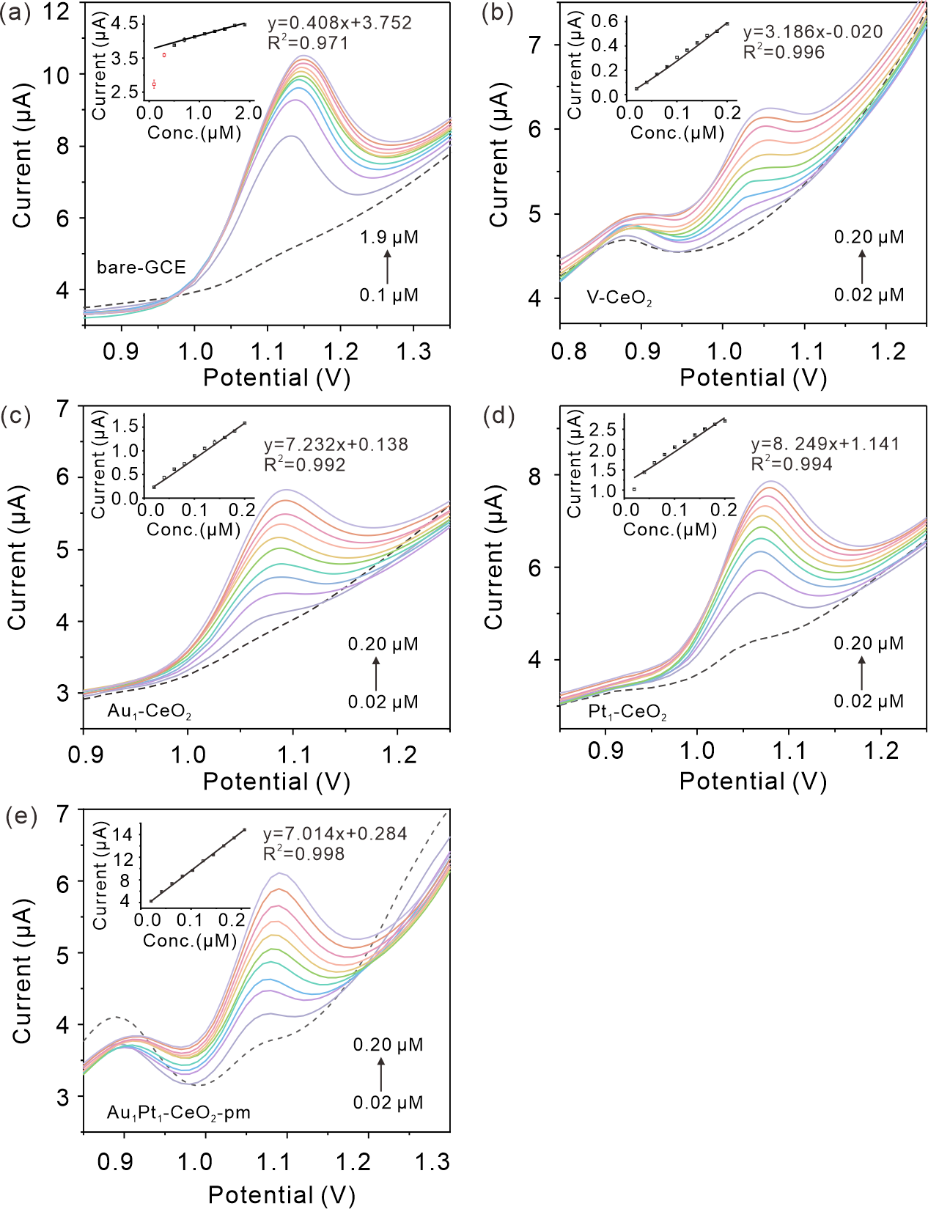


**Figure S19.** The electrochemical performance of NOR was measured by (a) bare-GCE and electrodes modified with (b) V-CeO_2_, (c) Au_1_-CeO_2_, (d) Pt_1_-CeO_2_ and (e) Au_1_Pt_1_-CeO_2_-pm. The linear correlation plots of current values versus NOR concentrations (inset).


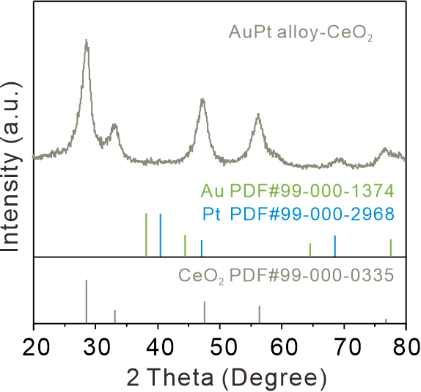


**Figure S20.** XRD spectra of AuPt alloy-CeO_2_.


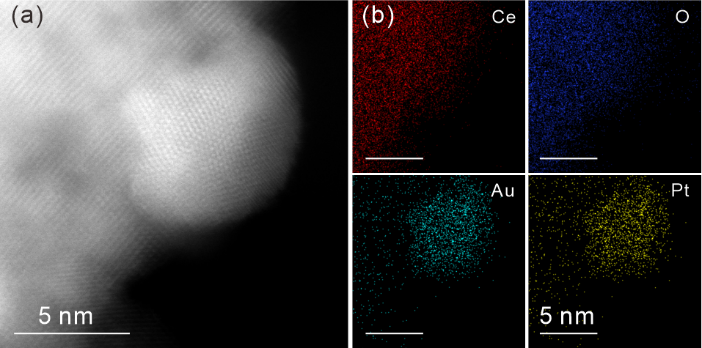


**Figure S21**. (a) HAADF-STEM images of AuPt alloy-CeO_2_. (b) Elemental mapping of Ce, O, Au, and Pt corresponding to (a).


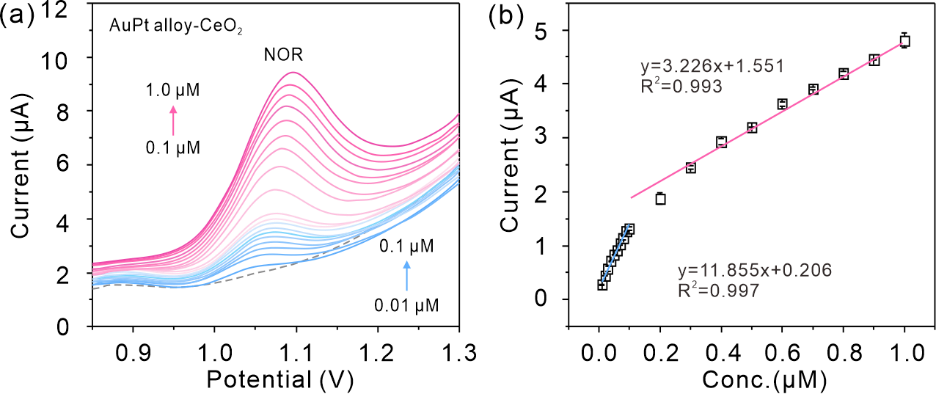


**Figu****re S22.** The electrochemical performance of AuPt alloy-CeO_2_ toward NOR. (a) SWV response signals of AuPt alloy-CeO_2_ toward NOR at extremely low concentration (0.01-0.10 μM, 10 steps with 0.01 μM intervals) and low concentration (0.10-1.0 μM, 10 steps with 0.10 μM intervals). (b) Linear correlations between peak currents and NOR concentrations of AuPt alloy-CeO_2_ for NOR detection in the range of 0.01-0.10 μM (R^2^ = 0.997) and 0.10-1.0 μM (R^2^=0.993), repeated 3 times.


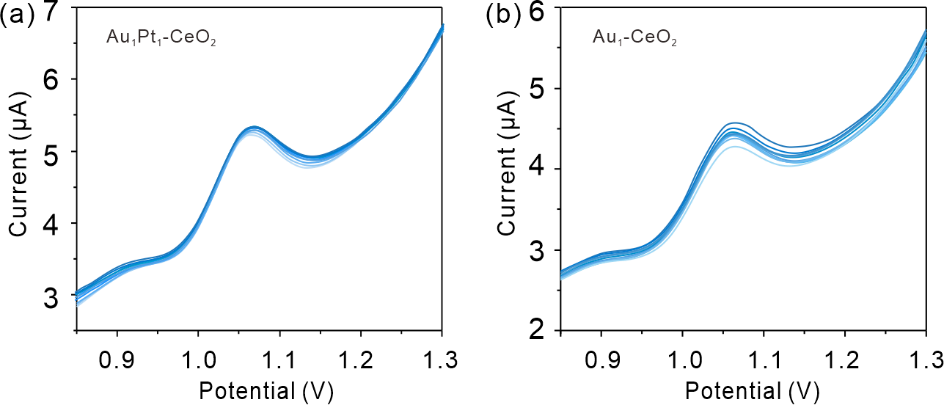


**Figure S23.** 10 continuous electrochemical experiments of (a) Au_1_Pt_1_-CeO_2_ and (b) Au_1_-CeO_2_ toward 0.05 μM NOR.


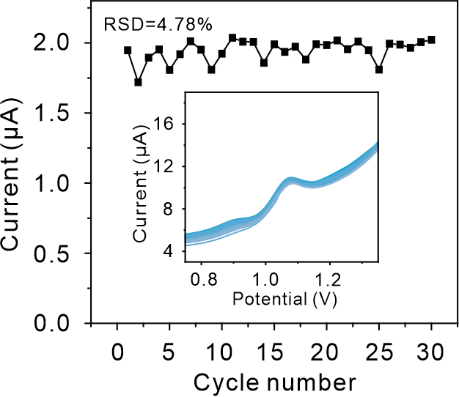


**Figure S24.** Stability experiments of Au_1_Pt_1_-CeO_2_/GCE toward 0.5 μM NOR in 0.1 M HAc-NaAc buffer solution.


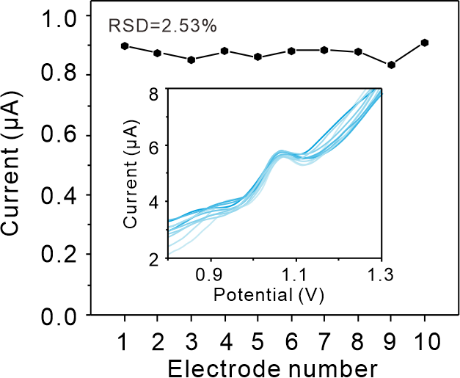


**Figure S25.** Robust reproducibility experiments of Au_1_Pt_1_-CeO_2_ toward 0.10 μM NOR demonstrate excellent repeatability.

To systematically evaluate the sensing performance of the Au_1_Pt_1_-CeO_2_/GCE, reproducibility was investigated under optimized catalysis conditions. 10 independently prepared Au_1_Pt_1_-CeO_2_/GCE underwent SWV in 0.10 μM NOR-containing electrolyte. As shown in Figure S25, the oxidation peak currents near +1.08 V exhibit little variation with a relative standard deviation (RSD) of 2.53% (< 5%), confirming the excellent reproducibility of the electrodes.


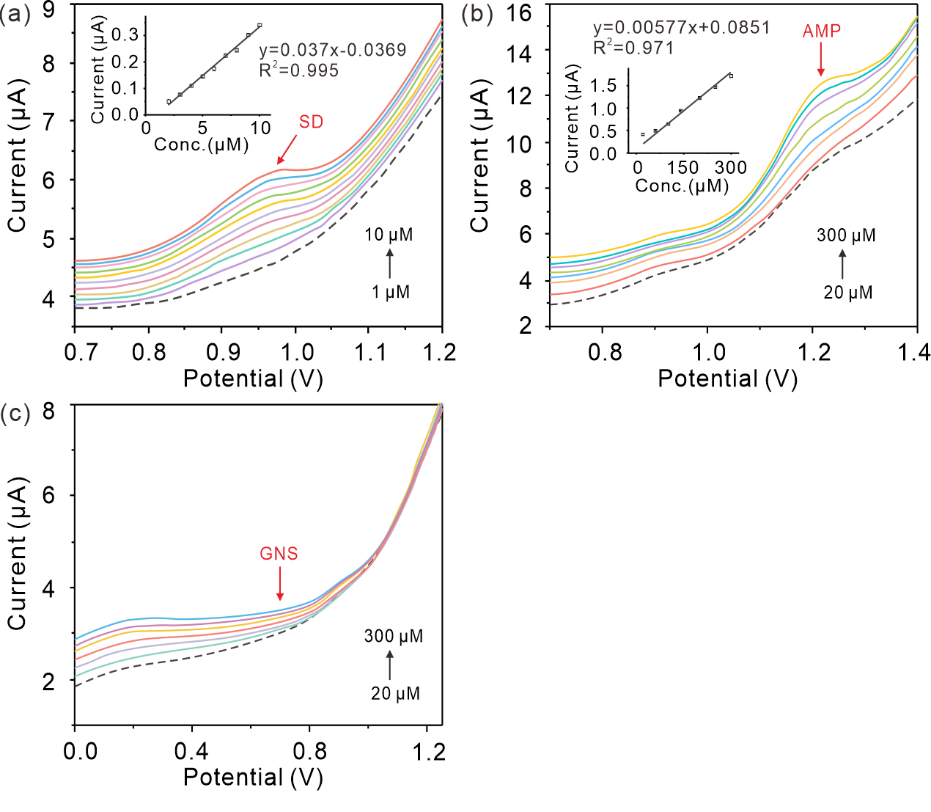


**Figure S26.** SWV response and linear equation of Au_1_Pt_1_-CeO_2_/GCE to (a) sulfadiazine (SD, sulfonamide) in the range of 1 to 10 μM (insert), (b) ampicillin (AMP, β-lactam) in the range of 20 to 300 μM (insert), (c) gentamicin sulphate (GNS, aminoglycoside) in the range of 20 to 300 μM.

Selective experiments on representative antibiotics for common antibiotic pollutants in real aqueous environments were carried out to evaluate the electrochemical response of different antibiotics *via* SWV. The results presented in Figure S26 show that Au_1_Pt_1_-CeO_2_ displays different electrochemical response peaks for SD, AMP, and GNS at the potentials of approximately +1.0 V, +1.25 V, and +0.7 V, respectively, which indicate that Au_1_Pt_1_-CeO_2_ is effective in realizing selective catalysis of NOR. Furthermore, the sensitivity of Au_1_Pt_1_-CeO_2_ for NOR is significantly higher than that of SD (0.037 μA μM^-1^) and AMP (0.0058 μA μM^-1^), which is nearly 366 and 2366 times higher than that of the latter two, respectively, while there is almost no current signal for GNS. The above results indicate that Au_1_Pt_1_-CeO_2_ has remarkable selectivity and superior sensitivity toward NOR.


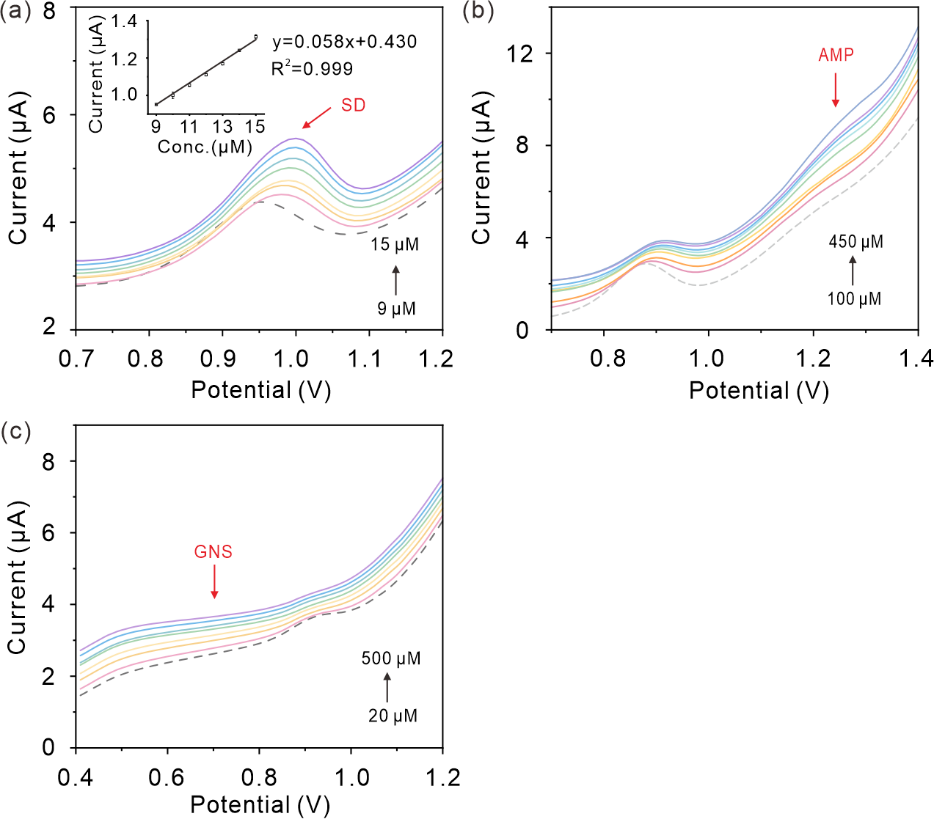


**Figure S27.**SWV responses of Au_1_-CeO_2_/GCE toward (a) SD (9-15 μM), (b) AMP (100-450 μM), and (c) GNS (20-500 μM). Insets show the corresponding calibration plots of peak current versus concentration with linear fits.

When only Au single-atom sites are present, Au_1_-CeO_2_/GCE tends to exhibit response characteristics dominated by single-center interactions. Consequently, molecules like SD, which primarily contain nitrogen-containing groups, can still generate a certain signal intensity, determined mainly by the direct contribution from a single Au site. Correspondingly, LOD of SD reflects its recognition capability under single-center interactions, without synergistic adsorption from adjacent sites. For AMP, despite its richer functional groups including carboxyl and amino groups, it often struggles to simultaneously satisfy the geometric constraints and electronic matching required for multi-site interactions at a single metal site, resulting in limited signal enhancement. GNS similarly exhibits weak response, primarily influenced by its solvation and diffusion characteristics.


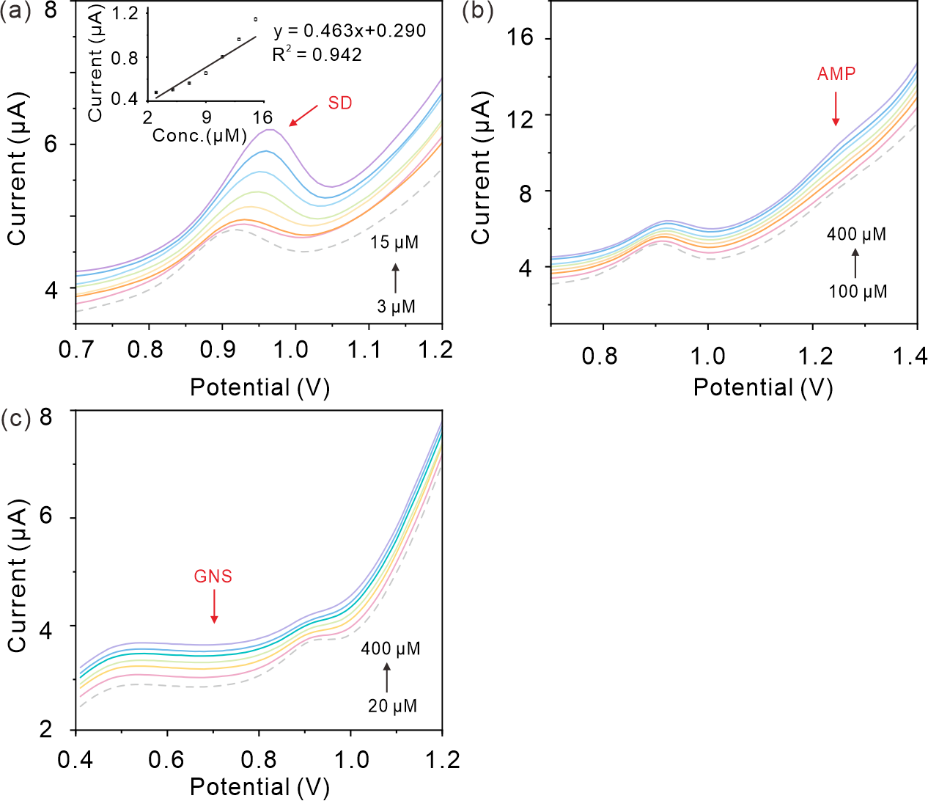


**Figure S28.** SWV responses of Pt_1_-CeO_2_/GCE toward (a) SD (3-15 μM), (b) AMP (100-400 μM), and (c) GNS (20-400 μM). Insets show the corresponding calibration plots of peak current versus concentration with linear fits.

Pt_1_-CeO_2_ exhibits a tendency to yield stronger signals for SD, suggesting Pt sites may be more prone to interacting with nitrogen-containing groups. Consequently, SD detection sensitivity is relatively higher, with a lower detection limit than Au_1_-CeO_2_. Due to the limitation of the active site, the enhancement in electrochemical response toward AMP is quite constrained. These results collectively demonstrate that single-metal sites are insufficient to provide synergistic multi-center active configurations to reduce selectivity toward NOR.


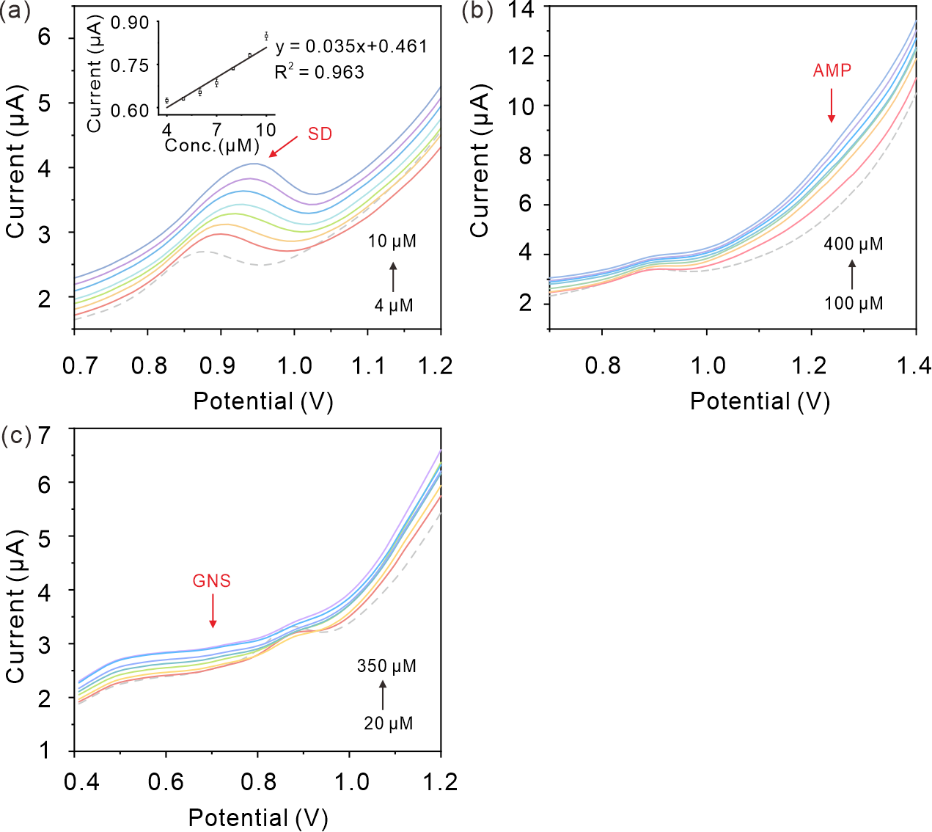


**Figure S29.** SWV response and linear equation of AuPt alloy-CeO_2_/GCE to (a) SD in the range of 4 to 10 μM (insert), (b) AMP in the range of 100 to 400 μM, (c) GNS in the range of 20 to 350 μM.

The overall weak signal characteristic of alloy samples may arise from the averaging of distinct site properties. When Au and Pt coexist in a more metallic state, the differentiated roles of local sites become harder to preserve, thereby hindering the formation of multi-interaction adsorption configurations with distinct geometric and electronic matching. In SD electrochemical testing, its LOD also fails to demonstrate superiority, reflecting the inability of the alloy surface to provide more recognizable local interaction differences for nitrogen-containing groups. This result further suggests that the decisive factor is not the simultaneous presence of both metals, but rather their stabilization in adjacent positions on the oxide surface, where they form differentiated interactions. Only then are they more likely to provide local binding configurations that can be specifically accessed by target molecules.


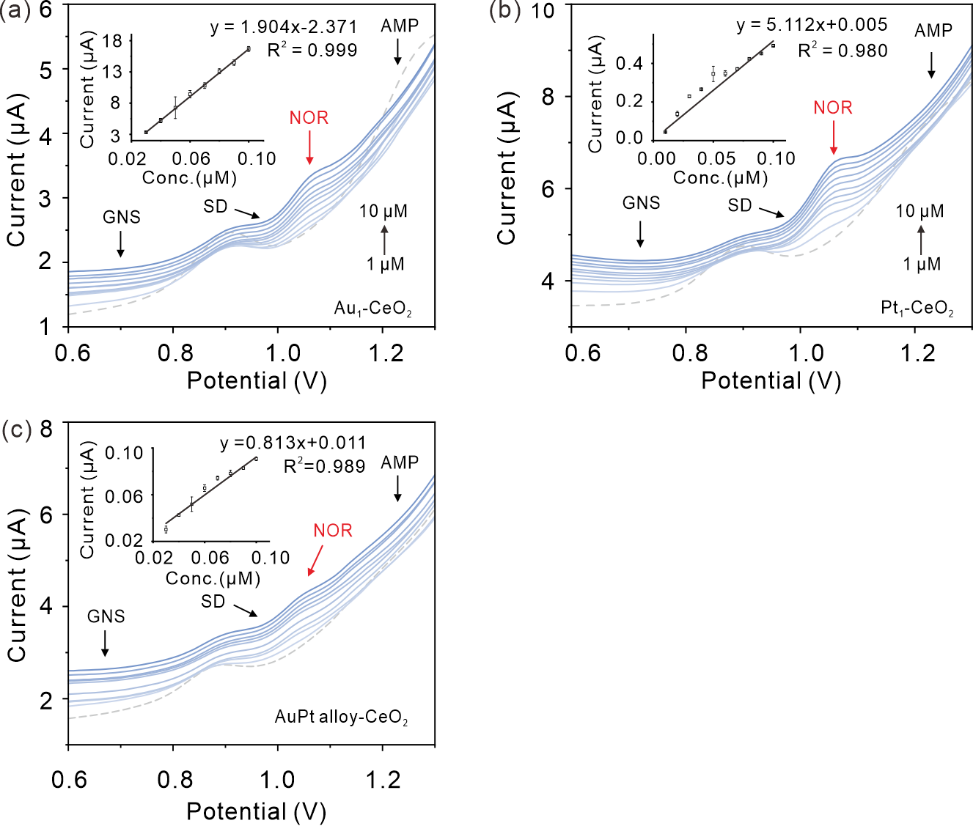


**Figure S30.** Anti-interference experiments of 0.01-0.10 μM NOR were carried out using (a) Au_1_-CeO_2_, (b) Pt_1_-CeO_2_, and (c) AuPt alloy-CeO_2_ in presence of 1.0 μM SD, AMP, and GNS, representative antibiotics form distinct pharmacological classes. The insets show the corresponding linear correlations between the peak currents and NOR concentrations.

As shown in Figure S30, when NOR is quantified under a competitive background containing 1 μM each of SD, AMP, and GNS, the NOR signal intensity exhibited by Au_1_-CeO_2_ and Pt_1_-CeO_2_ was significantly weaker than that of Au_1_Pt_1_-CeO_2_ (Figure 3g). This indicates that coexisting antibiotics can effectively occupy available single-metal sites, thereby competing with NOR. In the AuPt alloy-CeO_2_, the NOR signal further diminishes, consistent with the notion that alloying tends to homogenize the surface site environment, weakening the site differentiation required for molecular discrimination under multicomponent conditions.


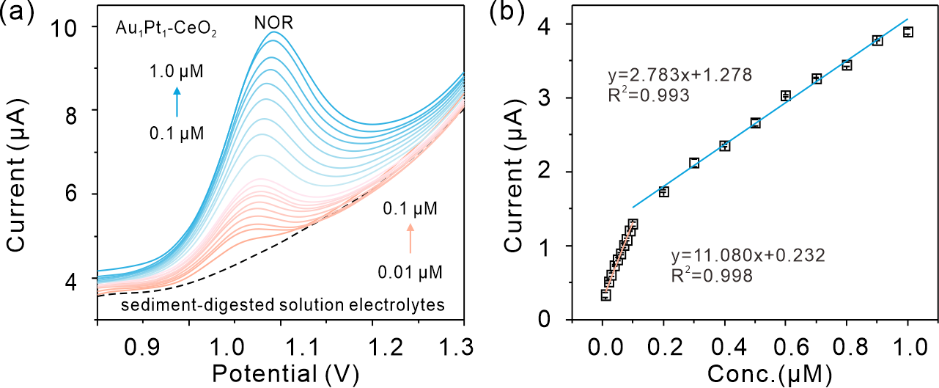


**Figure S31.** Complex environmental matrix samples catalysis performance of Au_1_Pt_1_-CeO_2_/GCE for NOR. SWV response of Au_1_Pt_1_-CeO_2_ to NOR in sediment-digested solution electrolytes. The catalysis was performed in extremely low (0.01-0.10 μM, R^2^=0.998) and low (0.10-1.0 μM, R^2^=0.993) concentration ranges, each with 10 data points measured in triplicate at uniform intervals. The corresponding linear equations are shown in (b), confirming the robust anti-interference performance of Au_1_Pt_1_-CeO_2_ in complex matrices.

The collected Suzhou Creek sediment samples underwent pre-treatment *via* acid digestion and microwave digestion to obtain the sediment samples for testing. These samples contained organic harmful pollutants, such as dioxin and chlorinated organic pollutants, as well as common pollutants and heavy metals present in Suzhou Creek water, which served as interferences. Considering the strong acidity of the sediment-digested samples, 8.0 g L^-1^ 100 μL sediment samples were added into the electrolyte to realize electrochemical catalysis under the interference of sediment-digested samples. Similarly, the NOR standard was used for the detection. As shown in Figures S31a-b, the detection sensitivity of Au_1_Pt_1_-CeO_2_/GCE was as high as 11.08 μA μM ^-1^, which is lower than that of the river water environment, which probably due to the more complex composition of the sediment but it still can reach the practical detection limit of 0.01 μM, and has a good linear relationship between the NOR concentrations and the SWV current values. All these show that Au_1_Pt_1_-CeO_2_/GCE has promising feasibility for application in a real water environment.


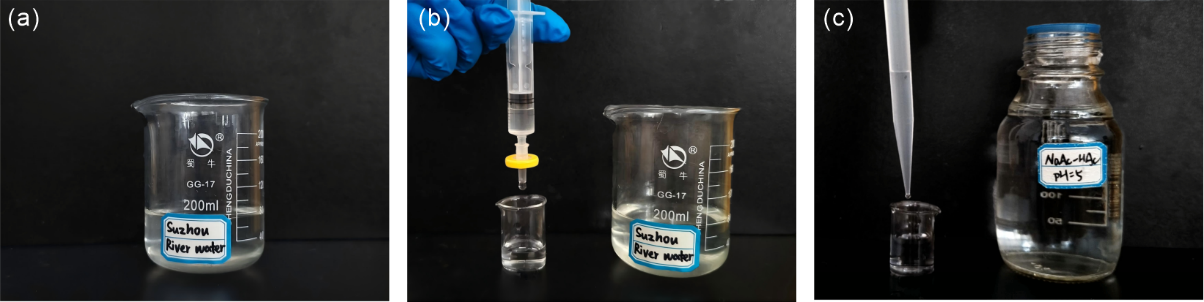


**Figure S32.** (a) Real water samples from Suzhou Creek, (b) The water sample of 1 mL Suzhou Creek was filtered into a beaker, (c) The process of adding 9 mL HAc-NaAc buffer solution to the real water sample. 1 mL sample of Suzhou Creek water was taken with a syringe and filtered through a 0.22 μm water filter tip. The purpose of the 9 mL HAc-NaAc buffer was to enhance the conductivity of the solution.


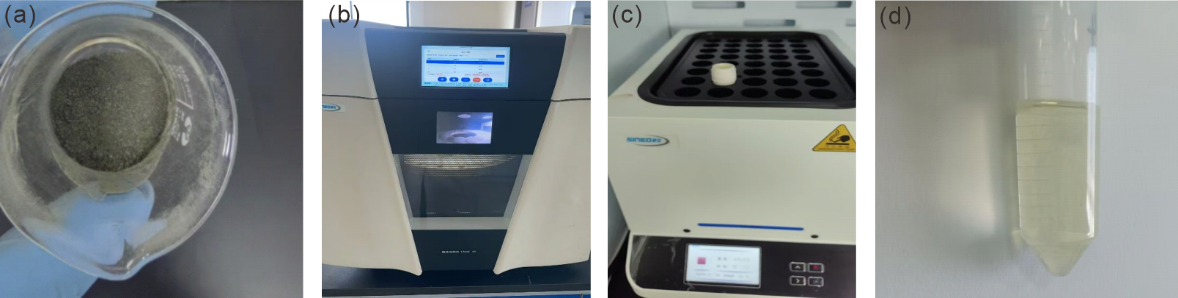


**Figure S33.** (a) Real river sediment samples from Suzhou Creek after drying treatment, (b) The process of using a TANK40 closed-vessel microwave digestion system with a mixture of (aqua regia and HF) to digest sediment samples. (c) A TK40 acid evaporation system was used to remove excess acid left by step (b). (d) Final sediment digested solution after volume adjustment.


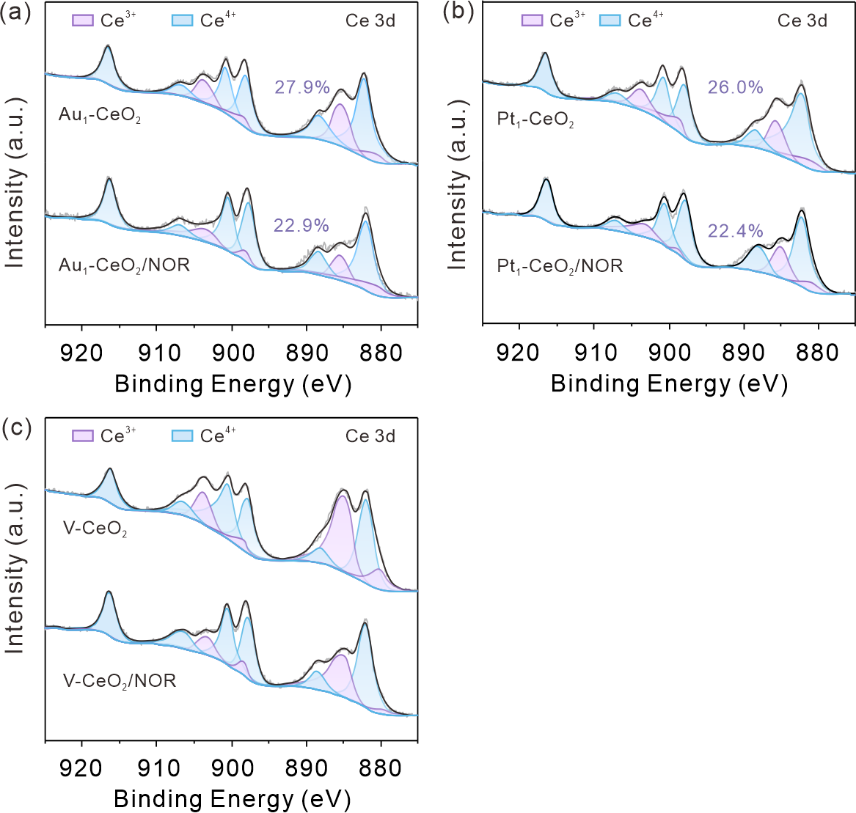


**Figure S34.** Comparison diagram of Ce 3d XPS before and after adsorption of NOR by (a) Au_1_-CeO_2_, (b) Pt_1_-CeO_2_, and (c) V-CeO_2_, the numbers in the figure represent the percentage amount of Ce^3+^.

The Ce 3d XPS of V-CeO_2_ adsorbed NOR is seen to have a significant decrease in the percentage of Ce^3+^ (Figure S34c), which indicates that Ce^3+^ is highly likely to undergo electron transfer to NOR and be converted to Ce^4+^. From the Ce 3d XPS plots of SACs after adsorption of NOR (Figures S34a, b), it can be found that the conversion of Ce^3+^ to Ce^4+^ is less efficient compared to V-CeO_2_, which may be due to the presence of a bridging structure of M-O-Ce (M=Au, Pt) in the SACs. This structure creates an electronic buffer region during the charge transfer process.


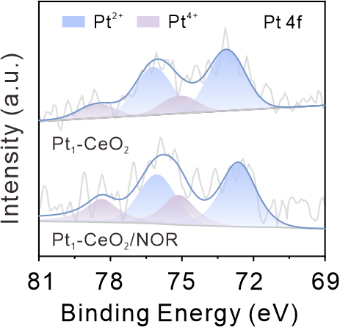


**Figure S35.** Comparison diagram of Pt 4f XPS before and after adsorption of NOR by Pt_1_-CeO_2_.


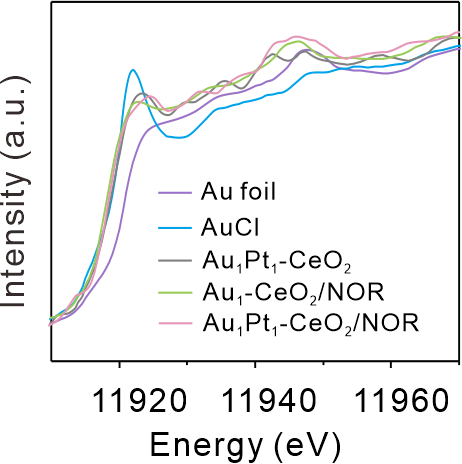


**Figure S36.** Normalized Au L_3_-edge XANES spectra of Au foil, AuCl, Au_1_Pt_1_-CeO_2_, and Au_1_Pt_1_-CeO_2_/NOR, where the Au valence state changes indicate an interaction between Au and Pt.


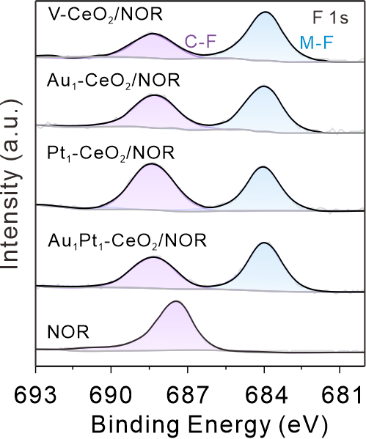


**Figure S37.** XPS of F 1s in V-CeO_2_/NOR, Au_1_-CeO_2_/NOR, Pt_1_-CeO_2_/NOR, Au_1_Pt_1_-CeO_2_/NOR, and NOR indicates that F is adsorbed on the samples in the form of M-F.

As shown in the F 1s XPS spectra after NOR adsorption, the newly emerged M-F signal in V-CeO_2_ exhibits the largest integrated area, suggesting strong interactions between the F of NOR and the defect-rich surface. Upon the introduction of single-atom Au or Pt, the M-F peak area decreases, whereas a significant increase is observed in Au_1_Pt_1_-CeO_2_. These variations imply distinct adsorption configurations. Combined with the O 1s XPS spectra (Figure S41), it can be inferred that NOR molecules predominantly bind to oxygen vacancies in V-CeO_2_, while the introduction of AuPt dual-atom sites alters the adsorption pathway, weakening the role of oxygen vacancies and promoting metal sites as the dominant adsorption centers.


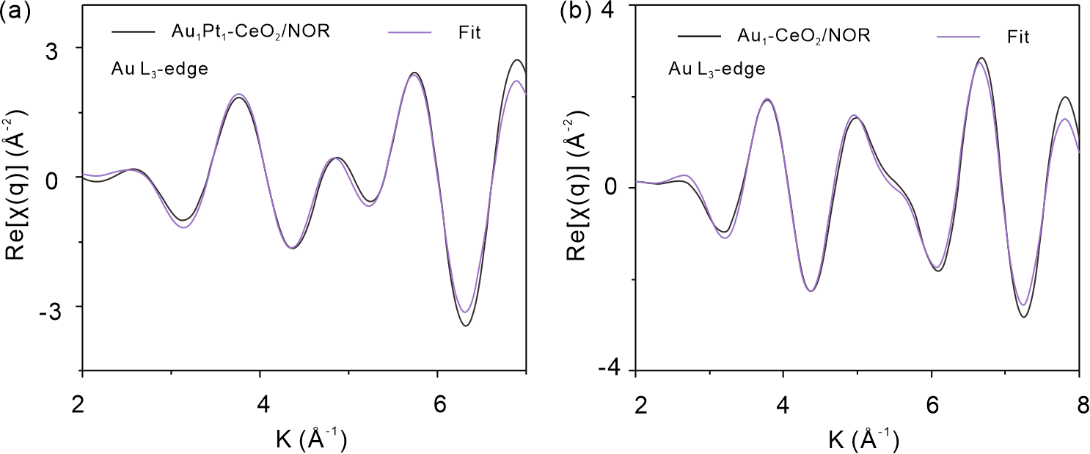


**Figure S38.** The corresponding k^3^χ(k) oscillations of (a) Au_1_Pt_1_-CeO_2_/NOR and (b) Au_1_-CeO_2_/NOR in Figure 4e, f.


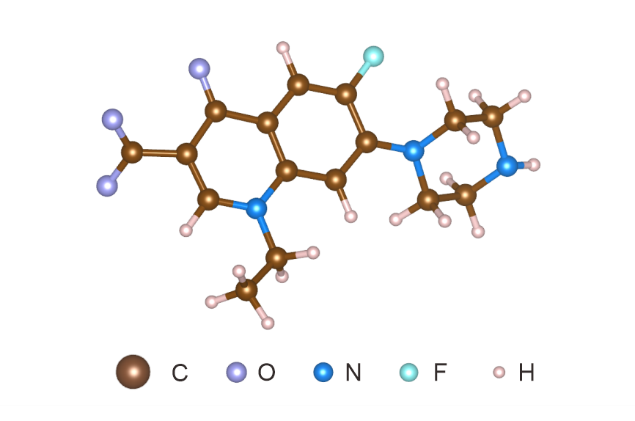


**Figure S39.** The optimized configuration of NOR.


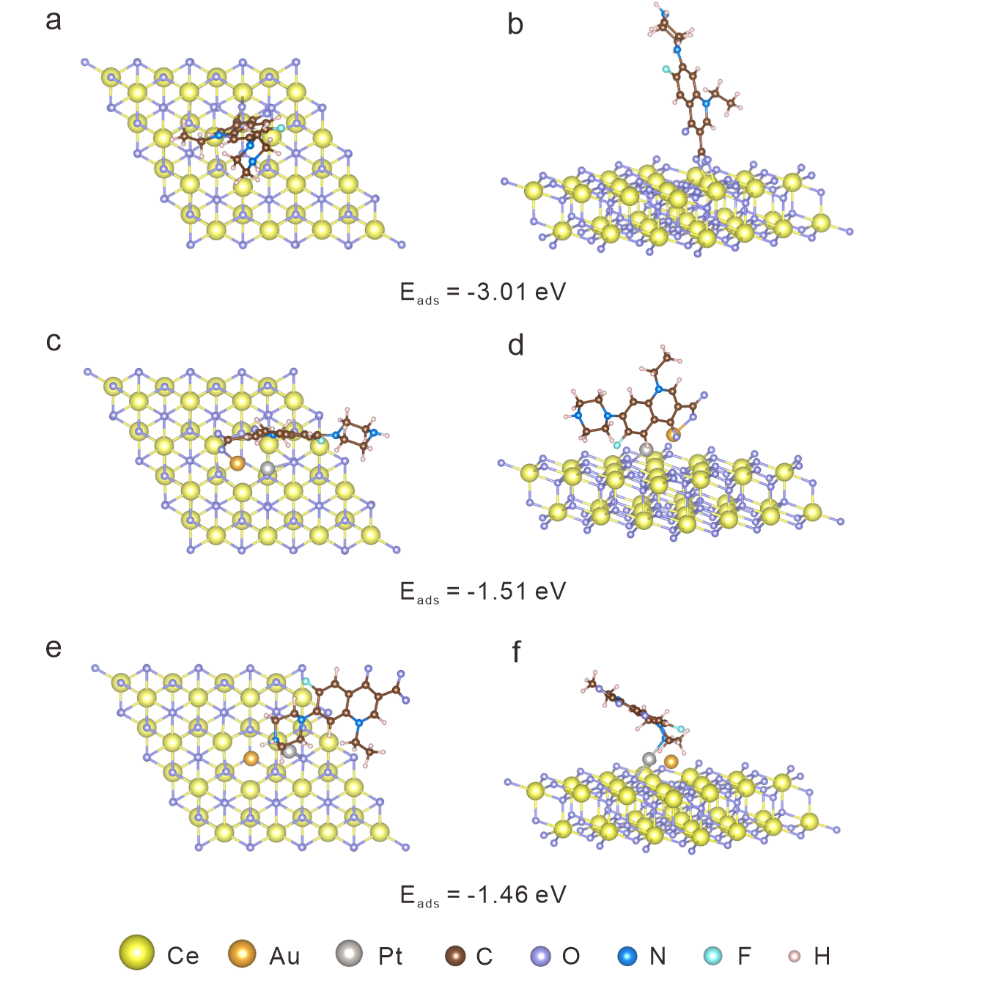


**Figure S40.** The optimized configurations and corresponding formation energy of (a, b) V-CeO_2_, (c, d) Au_1_Pt_1_-CeO_2_/NOR (where the carboxyl O in NOR mainly adsorbs to Au sites, the carbonyl oxygen mainly adsorbs to Pt sites), (e, f) Au_1_Pt_1_-CeO_2_/NOR (where the amine N in NOR mainly adsorbs to Pt sites).

NOR primarily adsorbs on V-CeO_2_ *via* oxygen vacancies. Two possible adsorption configurations exist in the presence of AuPt atoms. In configuration Figure S40 (c, d), oxygen-containing groups in NOR (-COOH and -C=O) bond with Au atoms, with the O atom in -C=O primarily interacting with Pt atoms. F atoms adsorb onto Ce. In configuration e, f, besides F atoms adsorbing onto Ce, the amino group bonds with Pt atoms.

_
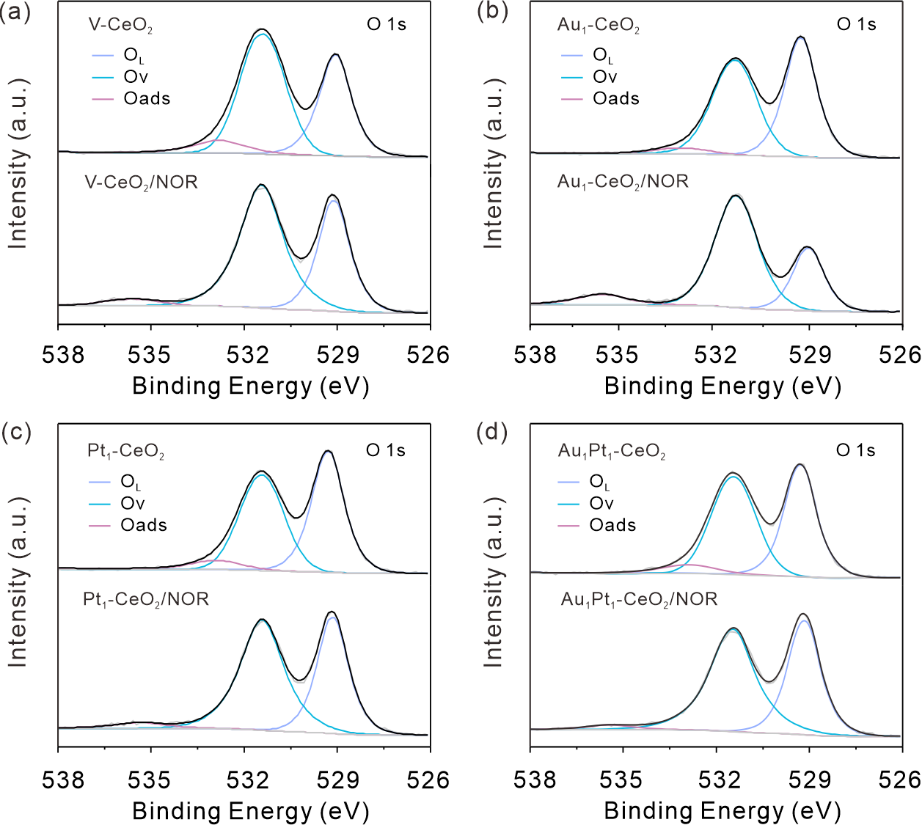
_

**Figure S41.** Comparison diagram of O 1s XPS before and after adsorption of NOR by (a) V-CeO_2_, (b) Au_1_-CeO_2_, (c) Pt_1_-CeO_2_, and (d) Au_1_Pt_1_-CeO_2_.

Compared with the V-CeO_2_ loaded with Au/Pt, the oxygen vacancy peak area of Figure S41(a) has decreased, which shows that the main adsorption behavior of V-CeO_2_ is for Ov adsorption.


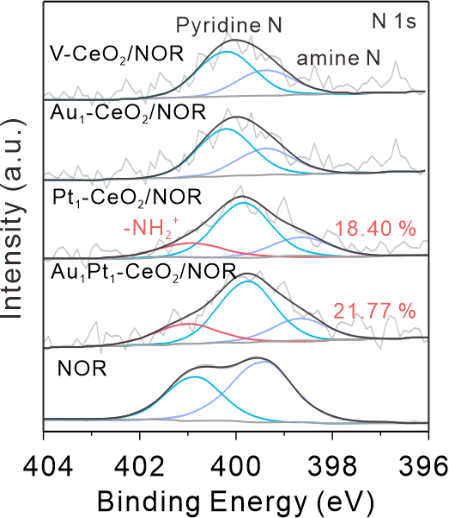


**Figure S42.** XPS of N 1s in V-CeO_2_/NOR, Au_1_-CeO_2_/NOR, Pt_1_-CeO_2_/NOR, Au_1_Pt_1_-CeO_2_/NOR, and NOR. The numbers in the figure represent the percentage amount of -NH_2_^+^.

Analysis of the N 1s XPS spectra reveals the presence of protonated amine groups (-NH_2_^+^), which are exclusive to Pt-containing systems and most pronounced in Au_1_Pt_1_-CeO_2_. It suggests that Pt exhibits a preferential interaction with -NH_2_^+^ groups. The bonding information of the metal active sites with NOR was further analyzed.


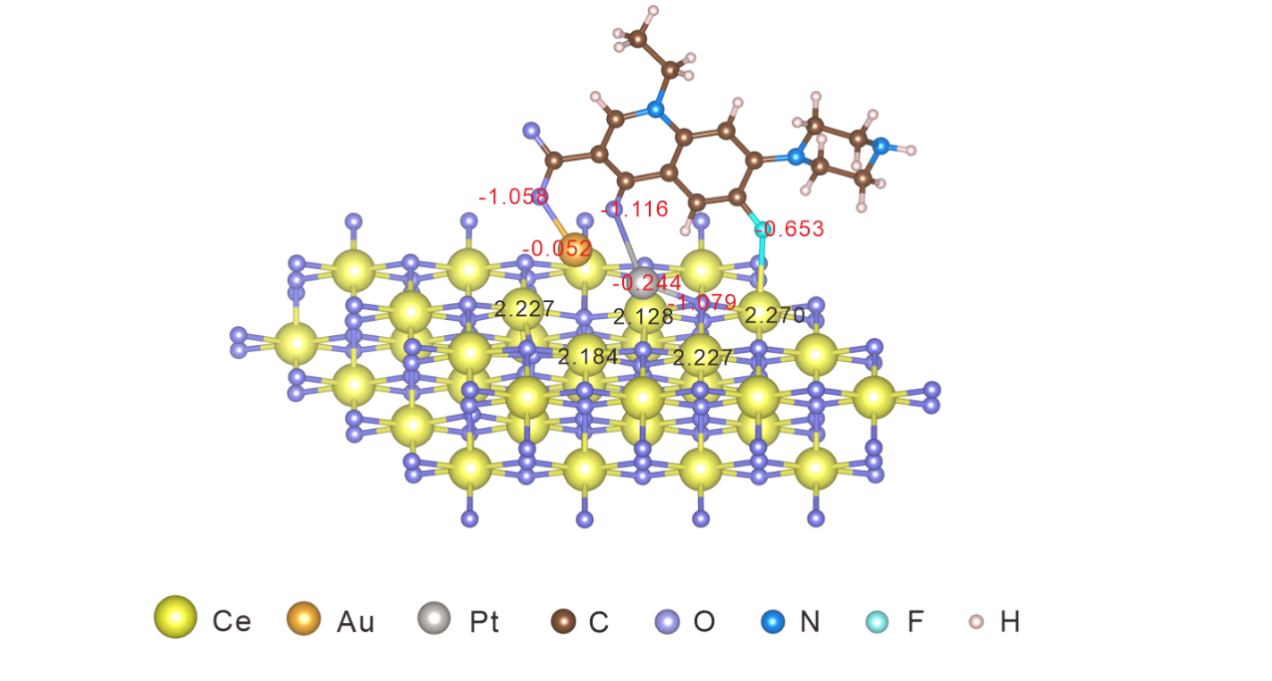


**Figure S43.** The Bader charges of Au_1_Pt_1_-CeO_2_/NOR (the yellow, orange, gray, brown, purple, blue, cyan, and pink balls represent the Ce, Au, Pt, C, O, N, F, and H atoms, respectively).

Figure S43 elucidates the details of interfacial charge transfer. The positive Bader charge on Ce in Au_1_Pt_1_-CeO_2_/NOR indicates that Au and Pt extract electrons from CeO_2_ *via* oxygen bridges. The Bader charge values for the carboxyl oxygen and carbonyl oxygen in NOR are -1.058 and -1.116 |e|, suggesting that electrons are predominantly transferred from Au and Pt atoms to oxygen.^[14]^ The negative Bader charge of F confirms the formation of a tridentate chelate coordination adsorption configuration.


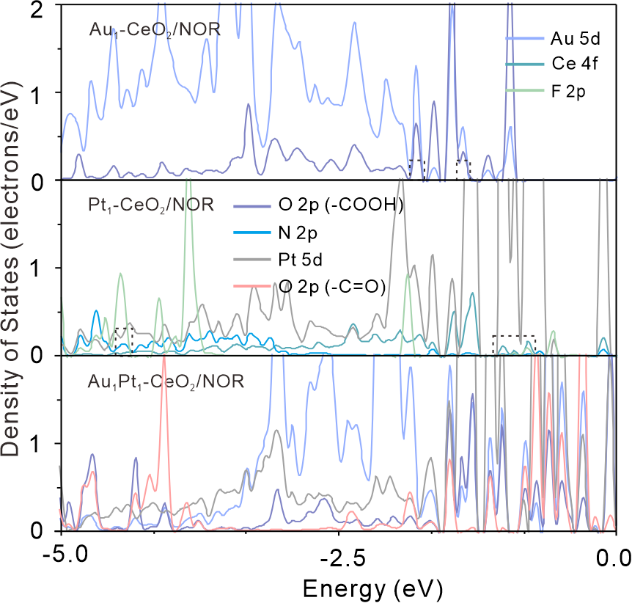


**Figure S44.** Comparison of PDOS diagram of Au_1_-CeO_2_/NOR, Pt_1_-CeO_2_/NOR, and Au_1_Pt_1_-CeO_2_/NOR exhibits well overlapped peaks for Au and carboxyl oxygen, Pt and carbonyl oxygen, and Ce and F atoms in Au_1_Pt_1_-CeO_2_/NOR. (Ce and F peaks are aligned and outlined with dashed boxes)

Compared to Pt_1_-CeO_2_/NOR, the presence of Au drives the conversion of Pt from bonding with N to bonding with the more electronegative O atom.


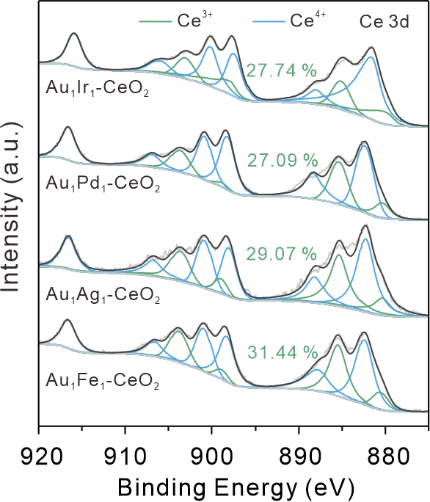


**Figure S45.** Comparison diagram of Ce 3d XPS by Au_1_Ir_1_-CeO_2_, Au_1_Pd_1_-CeO_2_, Au_1_Ag_1_-CeO_2_, and Au_1_Fe_1_-CeO_2_. The numbers in the figure represent the percentage amount of Ce^3+^.


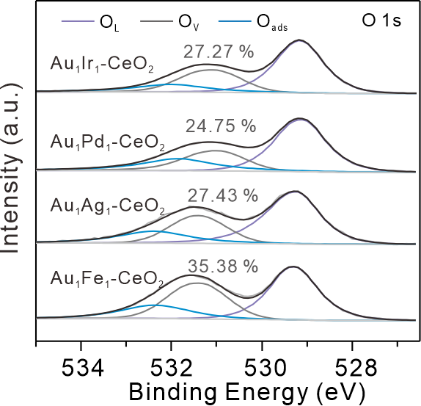


**Figure S46.** Comparison diagram of O 1s XPS by Au_1_Ir_1_-CeO_2_, Au_1_Pd_1_-CeO_2_, Au_1_Ag_1_-CeO_2_, and Au_1_Fe_1_-CeO_2_. The numbers in the figure represent the percentage amount of Ov.


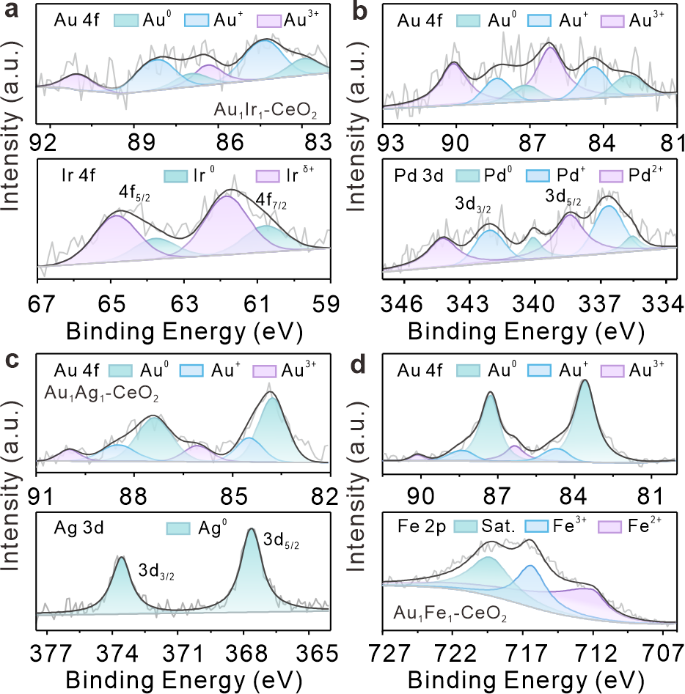


**Figure S47.** XPS of (a) Au 4f, Ir 4f in Au_1_Ir_1_-CeO_2_, and (b) Au 4f, Pd 3d in Au_1_Pd_1_-CeO_2_ reveal the configuration of Au single-atom dispersion, whereas (c) Au 4f, Ag 3d in Au_1_Ag_1_-CeO_2_, and (d) Au 4f, Fe 2p in Au_1_Fe_1_-CeO_2_ demonstrate Au agglomeration.

XPS analysis confirms that in Au_1_Ir_1_-CeO_2_ and Au_1_Pd_1_-CeO_2_, Au primarily exists as positively charged isolated single atoms, with most Pd and Ir atoms remaining atomically dispersed.^[15]^ Combining this with Ce^3+^ spectra, these results reveal that Pd and Ir can effectively regulate the reduction level of CeO_2_, providing more stable anchor sites for Au atoms (Figure S45). In contrast, Au_1_Ag_1_-CeO_2_ exhibits evident agglomeration of both Au and Ag, and Au_1_Fe_1_-CeO_2_ similarly shows Au agglomeration (Figure S47c, d). This may stem from the significant differences in the valence electron structure between Ag and Fe compare to PGMs. The 4*d*-filled electron orbital configuration of Ag results in its *d* orbital energy levels being low and stable. Besides, Fe, as a metal with strong oxygen affinity,^[16]^ coexists in both 2+ and 3+ oxidation states, exhibiting pronounced reducibility, which induces excessive reduction of the support and produces unstable oxygen vacancies that cannot stabilize isolated Au atoms (Figure S46).^[17]^


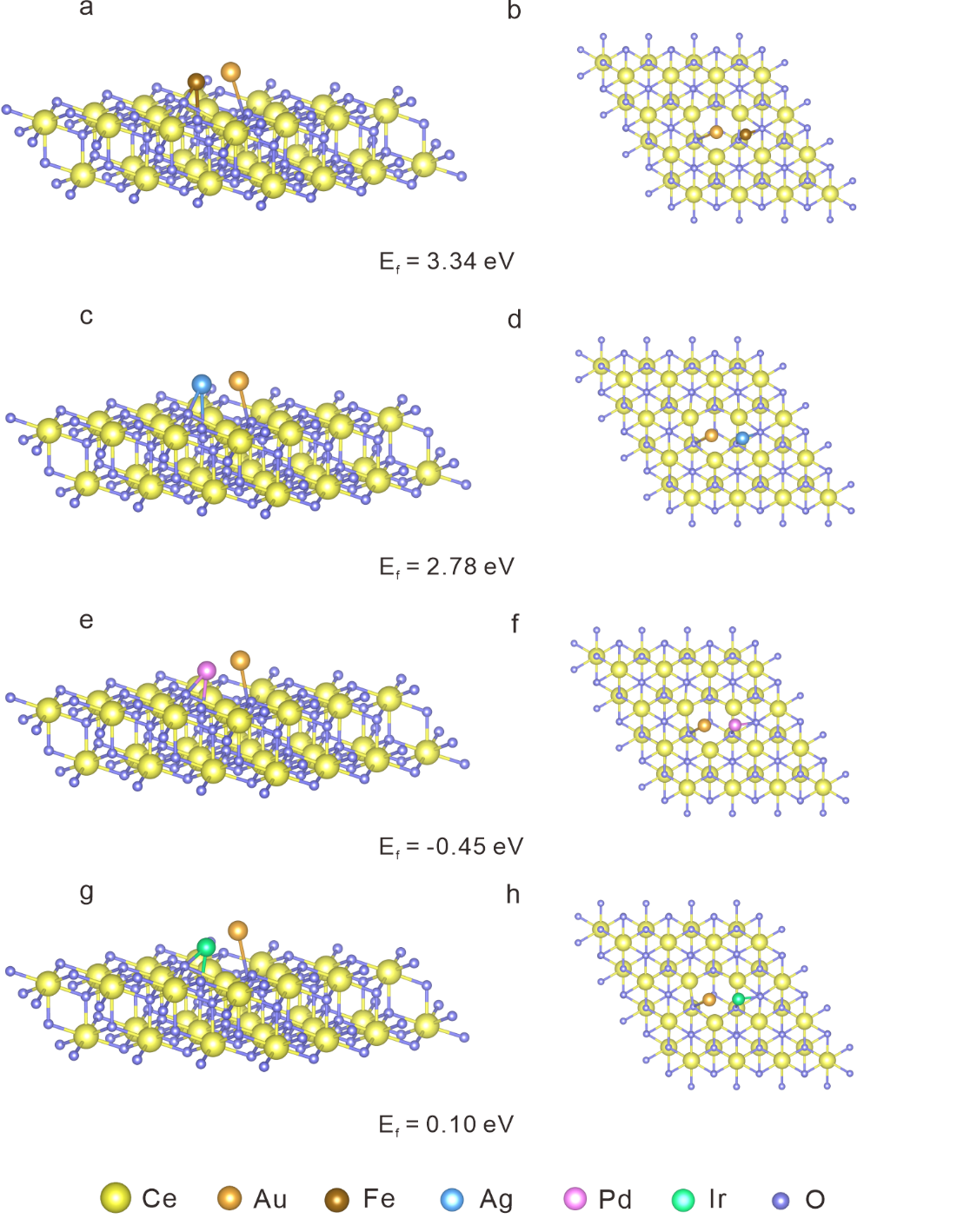


**Figure S48.** The optimized configurations and corresponding formation energy of (a, b) Au_1_Fe_1_-CeO_2_, (c, d) Au_1_Ag_1_-CeO_2_, (e, f) Au_1_Pd_1_-CeO_2_, and (g,h) Au_1_Ir_1_-CeO_2_.

1. **Tables.**

**Table** **S1.** ICP results of three different catalysts.

| Samples | Au (wt%) | Pt (wt%) |
| --- | --- | --- |
| Au_1_-CeO_2_ | 0.69 | - |
| Pt_1_-CeO_2_ | - | 0.75 |
| Au_1_Pt_1_-CeO_2_ | 0.79 | 0.76 |

**Table S2.** Pt L_3_-edge EXAFS fitting results of Au_1_Pt_1_-CeO_2_ sample by the ARTEMIS module of IFEFFIT.

| Sample | Path | CN | | | R (Å) | σ^2^ (10^-3^ Å^2^) | | | ΔE_0_ (eV) | R-factor | | S_0_^2^ | |
| --- | --- | --- | --- | --- | --- | --- | --- | --- | --- | --- | --- | --- | --- |
| Au_1_Pt_1_-CeO_2_ | Pt-O | | 8.47±0.39 | 2.27±0.01 | | | 0.2±0.01 | -29.5±0.10 | | | 0.0180 | 0.80 |  |

**Table S3.** Au L_3_-edge EXAFS fitting results of Au_1_Pt_1_-CeO_2_ sample by the ARTEMIS module of IFEFFIT.

| Sample | Path | CN | R (Å) | σ^2^ (10^-3^ Å^2^) | ΔE_0_ (eV) | R-factor | S_0_^2^ |
| --- | --- | --- | --- | --- | --- | --- | --- |
| Au_1_Pt_1_-CeO_2_ | Au-O1 | 0.71±0.01 | 1.95±0.02 | 5.0±0.01 | 13.88±0.01 | 0.0195 | 0.76 |
|  | Au-O2 | 3.50±0.01 | 2.77±0.02 | 3.5±0.01 | 13.88±0.01 |  |  |
|  | Au-O-Ce | 4.60±0.01 | 3.45±0.02 | 4.5±0.01 | 13.88±0.01 |  |  |

**Table S4.** Ce L_3_-edge EXAFS fitting results of V-CeO_2_, Au_1_-CeO_2_, Pt_1_-CeO_2_, and Au_1_Pt_1_-CeO_2_ samples by the ARTEMIS module of IFEFFIT.

| Sample | Path | CN | R (Å) | σ^2^ (10^-3^ Å^2^) | ΔE_0_ (eV) | R-factor | S_0_^2^ |
| --- | --- | --- | --- | --- | --- | --- | --- |
| V-CeO_2_ | Ce-O1 | 3.20±0.01 | 2.30±0.02 | 11.00±0.01 | 1.81±0.48 | 0.0197 | 0.75 |
|  | Ce-O2 | 1.66±0.01 | 3.15±0.02 | 0.13±0.01 | 1.81±0.48 |  |  |
|  | Ce-Ce | 2.48±0.11 | 3.81±0.02 | 2.51±0.01 | 1.81±0.48 |  |  |
| Pt_1_-CeO_2_ | Ce-O | 6.10±0.07 | 2.31±0.01 | 10.00±0.10 | 5.88±0.10 | 0.0107 |  |
|  | Ce-O-Pt | 1.66±0.18 | 3.19±0.04 | 0.40±0.01 | 4.51±0.10 |  |  |
|  | Ce-Ce | 4.46±0.15 | 3.84±0.01 | 3.00±0.10 | 4.51±0.10 |  |  |
| Au_1_-CeO_2_ | Ce-O | 4.10±0.12 | 2.29±0.12 | 9.80±0.20 | 4.67±0.20 | 0.0076 |  |
|  | Ce-O-Au | 1.80±0.14 | 3.17±0.03 | 8.00±0.10 | 4.67±0.20 |  |  |
|  | Ce-Ce | 2.80±0.10 | 3.82±0.01 | 3.00±0.10 | 4.67±0.20 |  |  |
| Au_1_Pt_1_-CeO_2_ | Ce-O | 4.74±0.01 | 2.29±0.01 | 11.00±0.10 | 3.52±0.79 | 0.0039 |  |
|  | Ce-O-Au | 2.10± 0.09 | 3.67±0.20 | 7.00±0.10 | 3.52±0.79 |  |  |
|  | Ce-O-Pt | 2.30±0.01 | 3.18±0.02 | 7.00±0.10 | 3.52±0.79 |  |  |
|  | Ce-Ce | 5.10±0.01 | 3.81±0.01 | 7.00±0.10 | 3.52±0.79 |  |  |

**Table S5.** Recovery experiments of NOR in real water samples.

| Sample | Added (μM) | Found (μM) | Recovery (%) | RSD (%) |
| --- | --- | --- | --- | --- |
| Suzhou Creek | 0.08 | 0.080 | 99.52 | 0.14 |
|  | 0.60 | 0.632 | 105.36 | 1.91 |

| Sample | Path | CN | R (Å) | σ^2^ (10^-3^ Å^2^) | | ΔE_0_ (eV) | R-factor | S_0_^2^ |
| --- | --- | --- | --- | --- | --- | --- | --- | --- |
| Au_1_-CeO_2_/NOR | Au-O | 0.61±0.01 | 1.99±0.10 | 10.08±0.01 | | 5.13±0.10 | 0.0135 | 0.76 |
|  | Au-Au | 6.33±0.01 | 2.85±0.02 | | 7.60±0.50 | 5.13±0.10 |  |  |
| Au_1_Pt_1_-CeO_2_/NOR | Au-O1 | 1.42±0.01 | 2.16±0.02 | | 6.00±0.01 | 1.17±0.01 | 0.0156 |  |
|  | Au-O2 | 5.39±0.01 | 2.47±0.03 | | 5.50±0.01 | 1.17±0.01 |  |  |
|  | Au-O-Ce | 9.50±0.01 | 3.49±0.03 | | 6.00±0.01 | 1.17±0.01 | 0.0156 |  |

**Table S6**. Au L_3_-edge EXAFS fitting results of Au_1_-CeO_2_/NOR and Au_1_Pt_1_-CeO_2_/NOR samples by the ARTEMIS module of IFEFFIT.

**Reference**

[1] P. Juhás, T. Davis, C. L. Farrow, S. J. L. Billinge, *J. Appl. Cryst.* **2013**, *46*, 560.

[2] C. L. Farrow, P. Juhas, J. W. Liu, D. Bryndin, E. S. Božin, J. Bloch, T. Proffen, S. J. L. Billinge, *J. Phys.: Condens. Matter* **2007**, *19*, 335219.

[3] X. Zhou, K. Li, Y. Lin, L. Song, J. Liu, Y. Liu, L. Zhang, Z. Wu, S. Song, J. Li, H. Zhang, *Angew. Chem.-Int. Edit.* **2020**, *132*, 13670.

[4] G. Kresse, J. Hafner, *Phys. Rev. B* **1993**, *48*, 13115.

[5] G. Kresse, J. Furthmüller, *Phys. Rev. B* **1996**, *54*, 11169.

[6] J. P. Perdew, K. Burke, M. Ernzerhof, *Phys. Rev. Lett.* **1996**, *77*, 3865.

[7] W. Kohn, L. J. Sham, *Phys. Rev.* **1965**, *140*, A1133.

[8] S. Grimme, J. Antony, S. Ehrlich, H. Krieg, *J. Chem. Phys.* **2010**, *132*, 154104.

[9] V. Wang, N. Xu, J.-C. Liu, G. Tang, W.-T. Geng, *Comput. Phys. Commun.* **2021**, *267*, 108033.

[10] K. Momma, F. Izumi, *J. Appl. Cryst.* **2008**, *41*, 653.

[11] L. M. Gámez, O. Resto, M. M. Martinez-Inesta, *MRS Proc.* **2014**, *1712*, mrss14.

[12] Y.-G. Wang, Y. Yoon, V.-A. Glezakou, J. Li, R. Rousseau, *J. Am. Chem. Soc.* **2013**, *135*, 10673.

[13] E. A. Derevyannikova, T. Y. Kardash, A. I. Stadnichenko, O. A. Stonkus, E. M. Slavinskaya, V. A. Svetlichnyi, A. I. Boronin, *J. Phys. Chem. C* **2019**, *123*, 1320.

[14] S. Liu, Y. Zhang, M. Wang, Y. Wei, Y. Wang, W. Chen, S. Mao, P. Guo, J. B. Ghasemi, J. Zhou, S. Zhang, X. Li, *Adv. Mater.* **2025**, *n/a*, e08693.

[15] Z. Li, H. Liu, A. Pang, S. Ji, X. Lu, Y. Zhang, C. Guo, L. Bai, J. H. Horton, Y. Wang, *Adv. Funct. Mater.* *n/a*, e05655.

[16] Y. Fan, M. Chu, H. Li, Z. Sun, D. Kong, J. Yao, G. Wang, Y. Wang, H.-Y. Zhu, *Small* **2024**, *20*, 2403804.

[17] X.-F. Yang, G.-P. Yi, P.-F. Lv, S.-J. Wen, Y.-P. Zhao, Z. Jing, Q. Wang, B. Li, P.-Y. Tang, *Electron* **2025**, *3*, e70011.
